# Antioxidant Capacity of Seminal Plasma and Its Association with Semen Quality and Dietary Antioxidant Intake

**DOI:** 10.3390/nu18152423

**Published:** 2026-07-24

**Authors:** Georgia Lagodonti, Dimitrios Kalompatsios, Panagiotis Varagiannis, Vassilis Athanasiadis, Stavros I. Lalas

**Affiliations:** 1 Department of Chemical Engineering, University of Western Macedonia, 50100 Kozani, Greece; georgia_lag@hotmail.com; 2 Department of Food Science & Nutrition, University of Thessaly, Terma N. Temponera Street, 43100 Karditsa, Greece; dkalompatsios@uth.gr (D.K.); pvaragiannis@uth.gr (P.V.); vaathanasiadis@uth.gr (V.A.)

**Keywords:** oxidative stress, seminal plasma, antioxidant capacity, lifestyle factors, FRAP assay, DPPH assay, male reproductive health

## Abstract

**Background/Objectives:** Male infertility is a growing public health concern, with lifestyle and dietary factors increasingly recognized as contributors to impaired sperm function. Oxidative stress plays a central role in sperm damage, yet the relationship between seminal antioxidant capacity, dietary antioxidant intake and semen quality remains unclear. This study investigated the association between seminal plasma antioxidant status, sperm quality parameters and dietary antioxidant intake, while accounting for key lifestyle factors. **Methods:** A total of 105 men (18–55 years) undergoing semen analysis were enrolled. Semen parameters were evaluated according to the WHO guidelines. Seminal plasma antioxidant capacity was assessed using the Ferric Reducing Antioxidant Power (FRAP) and DPPH radical scavenging assays. Dietary antioxidant intake was estimated using a validated food frequency questionnaire, and lifestyle factors (smoking, alcohol consumption, physical activity, supplement use) were recorded. Correlations and multivariable regression models were applied to examine associations between antioxidant indices and semen quality. **Results:** Seminal antioxidant capacity showed wide inter-individual variability (FRAP: 1.6–2171.4 μmol Trolox eq/L; DPPH: 28.5–3093.2 μmol Trolox eq/L). Dietary antioxidant intake demonstrated very weak correlations with semen parameters (r < 0.20). Regression analyses identified smoking and higher body weight as strong negative predictors of sperm concentration, total count and motility, whereas physical activity was positively associated with morphology and progressive motility. FRAP exhibited a mixed association with motility subtypes, while supplement use was positively associated with vitality. **Conclusions:** Lifestyle factors—particularly smoking, body weight and physical activity—were stronger determinants of semen quality than dietary antioxidant intake or seminal antioxidant indices. These findings highlight the importance of modifiable behaviors in male reproductive health and suggest that improving lifestyle habits may offer greater benefits than dietary antioxidant intake alone.

## 1. Introduction

Infertility is an emerging public health concern, with rising prevalence globally; recent estimates suggest that around one in six adults of reproductive age is affected, representing a substantial proportion of couples worldwide [1]. The male factor is directly or indirectly involved in up to 40–50% of cases, making the study of male reproductive health particularly important [2]. Recent epidemiological studies indicate a clear decline in sperm concentration and motility, highlighting the need to examine factors that are associated with sperm quality [3].

Beyond the biological dimension, male infertility could create several implications that may determine the quality of couple’s life (i.e., psychosocial and economic) and increase the use of reproductive-assisted technologies [4]. A variety of biological, environmental and social factors contribute to the rising incidence of infertility, including delayed childbearing, lifestyle changes, increased exposure to environmental pollutants, and metabolic disorders [5]. To that end, male fertility is no longer an isolated clinical issue, but is part of a broader field linked to of men’s general health and well-being. Understanding the factors associated with impaired sperm quality is crucial for developing prevention and early intervention strategies [6].

Sperm quality is correlated with several factors including genetics, environmental and behavioral factors [7]. Exposure to environmental pollutants combined with lifestyle factors (obesity, smoking, sedentary behavior), have been linked to deterioration in key sperm parameters [8]. Among modifiable factors, diet plays a particularly important role. Dietary patterns rich in fruits, vegetables, nuts, olive oil and other antioxidant-rich foods have been positively associated with sperm concentration, motility and morphology, whereas diets high in saturated fats and processed foods appear detrimental [9]. However, the underlying mechanisms through which diet is related to sperm function remain incompletely understood.

Oxidative stress is a key pathophysiological mechanism through which environmental and dietary factors correlate with sperm quality. It results from an imbalance between the production of reactive oxygen species (ROS) and the human antioxidant mechanisms responsible for neutralizing them [10]. While small amounts of ROS are necessary for normal sperm function, elevated levels are negatively associated with its quality and fertilizing capacity. Increased oxidative stress can lead to lipid peroxidation of the sperm cell membrane, adversely correlated with motility and morphology; parameters assessed during routine semen analysis [11]. Furthermore, ROS and their metabolites can damage sperm DNA, lipids and proteins, ultimately causing cell death [12]. A sufficient presence of antioxidant compounds in semen helps maintain the functional capacity of spermatozoa and positively correlates with key semen quality parameters, underscoring the importance of dietary antioxidant intake in male reproductive health. Semen antioxidant capacity could be associated with dietary antioxidant compounds and several lifestyle factors [13]. Several assays, including reducing capacity (FRAP) and radical scavenging methods (DPPH), have been used to quantify seminal antioxidant status, yet their relationship with semen quality and dietary antioxidant intake remains insufficiently explored. Correlating these assay results with semen quality parameters and dietary intake could identify how modifiable factors determine male fertility through oxidative stress [14].

Although previous studies have examined associations between dietary antioxidants and male fertility, findings remain inconsistent, partly due to methodological heterogeneity, limited sample sizes and inadequate adjustment for lifestyle confounders such as smoking, alcohol consumption, physical activity and supplement use. Moreover, few studies have directly compared different biochemical assays of seminal antioxidant capacity or evaluated their concordance.

The aim of this study was to investigate the relationship between seminal plasma antioxidant capacity, semen quality parameters and dietary antioxidant intake in a cohort of men undergoing semen analysis. A secondary objective was to assess the correlation of key lifestyle factors—including smoking, alcohol consumption, physical activity and supplement use—on semen quality. By integrating biochemical, dietary and lifestyle data, this study seeks to clarify the role of modifiable behaviors in male reproductive health and to identify potential targets for non-pharmacological intervention.

## 2. Materials and Methods

### 2.1. Chemicals and Reagents

Sperm morphology evaluation was performed using the Papanicolaou staining method according to the WHO guidelines. The staining procedure utilized ethanol for fixation, Harris hematoxylin for nuclear staining, Orange G-6 solution, and EA-50 stain for cytoplasmic differentiation. Hydrochloric acid and lithium carbonate solutions were used during the differentiation and bluing steps, respectively. All staining reagents were of analytical grade.

Sperm vitality was assessed using the FertiPro™ Vitality Kit (FertiPro N.V., Beernem, Belgium), following standard WHO-recommended procedures for distinguishing viable from non-viable spermatozoa based on membrane integrity.

Sperm concentration was determined using a WHO-standard semen dilution solution containing sodium bicarbonate, formaldehyde and distilled water, enabling sperm immobilization and accurate counting with a Neubauer hemocytometer.

All reagents used for antioxidant assays (FRAP and DPPH) were of analytical grade.

### 2.2. Instruments and Software

Semen analysis and microscopic evaluation were performed using an iScope microscope (IS.1153-PLPHi; Euromex Microscopen BV, Arnhem, The Netherlands) for the assessment of sperm concentration, motility, morphology and general semen characteristics according to the WHO guidelines.

Sample incubation and temperature stabilization were conducted using a Cultura M incubator (IVFtech ApS, Birkerød, Denmark). Homogenization and sample mixing were performed using an RSLAB-6PRO adjustable vortex mixer (RSLAB, Shanghai, China), ensuring standardized preparation of seminal plasma samples prior to antioxidant analysis. Glass microscope slides and coverslips were used for smear preparation and cytological staining.

Statistical analysis was performed using JMP Pro 16 (SAS Institute Inc., Cary, NC, USA).

### 2.3. Study Design

This cross-sectional study investigated the relationship between semen antioxidant status, semen quality and dietary antioxidant intake, while also examining the impact of lifestyle factors, including smoking, alcohol consumption, physical activity and dietary supplement use, on sperm function. Semen parameters were evaluated according to the WHO guidelines [15].

#### 2.3.1. Study Population

A total of 105 men aged 18–55 years undergoing semen analysis were recruited. No formal a priori sample size calculation was conducted, as this was an exploratory cross-sectional study utilizing a convenience sampling method. The overall sample size (n = 105) was similar to or surpassed that of other prior research studies [16,17,18] investigating similar correlations between antioxidant levels and semen quality indicators. Inclusion criteria included 2–5 days of sexual abstinence, ability to provide a semen sample and written informed consent. Exclusion criteria included genetic abnormalities, acute infections, chronic diseases that are associated with fertility and history of malignancy. Participant characteristics are presented in Supplementary Table S1.

#### 2.3.2. Semen Collection and Preparation

Semen samples were collected through masturbation into sterile containers and allowed to liquefy at 37 °C prior to analysis. All samples were analyzed within 30 min of collection.

#### 2.3.3. Semen Analysis

Semen analysis was performed according to the WHO guidelines [15] by qualified personnel. Semen volume, sperm concentration, motility, morphology and viability were assessed using standard laboratory methods [19]. Semen concentration was determined with a Neubauer hemocytometer, and motility was evaluated microscopically. Semen motility was divided in the fast progressive motility A (ProgA) and the slow progressive motility (ProgB). Each morphology was assessed using Papanicolaou staining under strict criteria and viability was determined with eosin–nigrosin staining [20].

Semen analysis was performed according to the WHO Laboratory Manual (6th Edition), using standardized procedures and internal quality control. Although conventional semen analysis remains the cornerstone of male fertility assessment, it does not evaluate functional sperm competence, oxidative stress, DNA integrity or fertilization potential. Therefore, semen parameters should be interpreted within the broader clinical context.

### 2.4. Assessment of Antioxidant Status

Following semen analysis, seminal plasma was isolated by means of centrifugation for antioxidant assessment. Total antioxidant capacity was evaluated using the iron-reducing [21] and radical scavenging methodologies [22] to examine both ion-reducing and radical scavenging capacities.

#### 2.4.1. Ferric Reducing Antioxidant Power (FRAP)

The total antioxidant capacity of the seminal plasma was determined using the FRAP method, which is based on the ability of the antioxidants in the sample to reduce ferric ions (Fe^3+^) to ferrous ions (Fe^2+^) in the presence of the chromogen ligand TPTZ (2,4,6-tripyridyl-*s*-triazine), forming a complex of intense blue color. Specifically, 50 μL of seminal plasma sample and 50 μL of FeCl_3_ solution (4 mM) were added to a test tube. The mixture was incubated for 30 min at 37 °C. Next, 900 μL of TPTZ solution (1 mM) was added, followed by a further 5 min of incubation at room temperature. A 5 min centrifugation at 10,000× *g* was required to settle the precipitates. The absorbance of the final mixture was measured spectrophotometrically at 620 nm. Three measurements were taken for each sample. A mixture containing 50 μL of distilled water, 50 μL of FeCl_3_ (4 mM) and 900 μL of TPTZ (1 mM) was used as a blank. The antioxidant capacity of the samples was expressed in Trolox equivalents, based on a reference curve (Table A1).

#### 2.4.2. DPPH Radical Scavenging Activity

The antioxidant activity of the seminal plasma was also assessed using the DPPH (2,2-diphenyl-1-picrylhydrazyl) method, which is based on the reduction in the stable DPPH free radical by antioxidant compounds in the sample. The reduction in the radical is accompanied by a change in the color of the solution from deep purple to yellowish. Initially, 25 μL of methanol and 975 μL of DPPH solution (100 μM) were added, and the initial absorbance at 515 nm was measured. Next, 25 μL of centrifuged seminal plasma sample and 975 μL of methanolic DPPH solution (100 μM) were added to a new tube. The mixture was incubated for 30 min in the dark at room temperature. After incubation, the absorbance was measured spectrophotometrically at 515 nm. Three measurements were taken for each sample. Antiradical activity (*A*_AR_) was examined using Trolox calibration curve, as shown in Table A1.

### 2.5. Dietary and Lifestyle Assessment

#### 2.5.1. Nutritional Assessment

Dietary intake was assessed using a semi-quantitative food frequency questionnaire (FFQ) adapted to the Greek population [23]. The questionnaire evaluated habitual intake of antioxidant-rich foods, including fruit, vegetables, nuts, olive oil, herbal teas and spices. Semi-quantitative FFQs are widely used in nutritional epidemiology to assess long-term dietary patterns and reported frequencies and portion sizes can be converted into daily gram weights and multiplied by nutrient composition values to estimate quantitative intake.

Body weight was categorized into predefined intervals (≤60, 61–70, 71–80, 81–90, and >90 kg) based on percentile cutoffs of the sample distribution to ensure adequate group sizes for statistical comparisons. Height measurements were not collected; therefore, body mass index (BMI) could not be calculated. As a result, body weight was used as the available anthropometric indicator and should be interpreted as a surrogate measure rather than a direct index of adiposity.

The collected data were used to examine the association between dietary antioxidant intake, semen antioxidant status and sperm quality. The questionnaire with thorough details can be found in Supplementary Table S2.

#### 2.5.2. Lifestyle Questionnaire

Participants also completed a lifestyle questionnaire assessing smoking status, alcohol consumption, physical activity and dietary supplement use. These variables were included in the statistical analysis to investigate their potential associations with semen quality, oxidative stress and the relationship between antioxidant intake and sperm parameters [24]. The lifestyle questionnaire was created using previously validated and established epidemiological frameworks examining modifiable risk factors for male infertility [25,26].

Smoking categories were defined according to daily cigarette use, following established epidemiological cutoffs (non-smoker, light, moderate, heavy). Alcohol intake categories (non-drinker, occasional, moderate, heavy) were determined according to the WHO standard drink units per week.

Physical activity was classified as ‘inactive’ (absence of regular exercise) vs. ‘minimally active’ (≥30 min of moderate-intensity activity on ≥3 days per week). This simplified classification was intentionally selected to reduce recall bias, improve participant compliance, and align with previous fertility studies employing binary activity metrics.

The use of dietary supplements was evaluated as a quaternary variable (never, monthly, weekly, daily). Supplement categories (multivitamins, folate, vitamin C, iron, omega-3 PUFAs, other antioxidants) were selected based on established evidence linking these micronutrients to reproductive physiology, oxidative balance and sperm function.

The questionnaire with thorough details can be found in Supplementary Table S3.

### 2.6. Statistical Processing

#### 2.6.1. Data Processing

Statistical analysis was conducted using JMP Pro 16 (SAS Institute Inc., Cary, NC, USA) to investigate the relationships between semen quality parameters, antioxidant status, dietary antioxidant intake and lifestyle factors. We introduced Oxygen Radical Absorbance Capacity (ORAC) for in vitro antioxidant evaluation of selected foods by means of evaluating only a theoretical antioxidant capacity in human body. Continuous variables were assessed for normality and transformed where necessary, with log10 transformation applied to sperm concentration, total sperm count, ORAC and FRAP values, and arcsin transformation applied to percentage semen parameters.

#### 2.6.2. Regression Models

Lifestyle variables, including smoking, alcohol consumption, physical activity and supplement use, were categorized for inclusion in the statistical models. Pearson’s correlation coefficients were calculated to assess associations between antioxidant indices (ORAC, FRAP, DPPH) and semen parameters. Linear regression models using the Standard Least Squares method were developed to evaluate the effects of demographic characteristics, lifestyle factors and antioxidant markers on semen quality. Model validity was assessed through residual diagnostics, including residual plots, studentized residuals, leverage plots and Bonferroni outlier testing.

#### 2.6.3. Correlation Analyses

Principal component analysis (PCA) was additionally performed to explore the overall structure of the dataset and identify correlations among semen parameters, demographic variables and lifestyle factors, while antioxidant indices were included as supplementary variables. A significant correlation between antioxidant consumption and sperm quality was expected a priori as a linear trend in the relationship between antioxidant consumption and the relevant semen quality. Data visualization techniques, including boxplots, heatmaps, PCA biplots and residual plots, were used to facilitate interpretation of the findings. Statistical significance was set at *p* < 0.05, with *p*-values between 0.05 and 0.10 considered indicative of trends. Post hoc power analysis, based on an effective sample size of approximately 100 participants, demonstrated limited power for detecting small correlations between antioxidant markers and semen quality parameters. However, the overall findings from the regression analyses and PCA suggested that lifestyle factors exerted a greater association with sperm quality than antioxidant indices, supporting the conclusion that antioxidant markers were not major predictors of semen quality in the present study.

## 3. Results

### 3.1. Antioxidant Capacity of Seminal Plasma

A large variation between individuals was observed in both antioxidant assays in the descriptive analysis (Table S1). A wide range in DPPH values was observed (28.5–3093.2 μmol Trolox eq/L), which indicated moderate heterogeneity. The results also showed significant variation in the FRAP technique among the participants (1.6–2171.4 μmol Trolox eq/L). It is worth mentioning that one individual (patient ID 3084) displayed abnormally high levels of both DPPH and FRAP assays, indicating an outlier. This could also indicate either a unique biological composition (such as abnormally high consumption of antioxidants in the dietary intake or genetic variant). The greater variation in the FRAP method could suggest that the two measures of semen antioxidant activity state may be different, but complementary.

### 3.2. Correlations Between Antioxidant Indices and Semen Parameters

Pearson correlation analysis between antioxidant intake indices (ORAC fruit, vegetables, nuts, cocoa, spices and ORAC total) and sperm quality parameters revealed extremely low correlation coefficients in all cases (r < 0.20). None of the individual ORAC variables showed a moderate or strong linear relationship with sperm motility, morphology, concentration, total count or viability, as shown in Table 1. This finding suggests that dietary antioxidant intake, as assessed by ORAC values, is not directly associated with THE WHO parameters in the present sample. The absence of significant correlations was also confirmed using subsequent multivariate and multidimensional analyses (regression models, PCA and heatmap), reinforcing the reliability of the result (vide infra).

### 3.3. Regression Models

Regression analyses identified lifestyle factors as the primary determinants of sperm quality. Increased body weight and smoking were associated with lower sperm concentration, total count and motility, whereas physical activity and supplement use showed beneficial effects on morphology, progressive motility and vitality. FRAP demonstrated a mixed effect depending on motility subtype. Model explanatory power ranged from R^2^ = 0.16 to 0.28 across semen parameters.

#### 3.3.1. Motility

The significant predictors, regression coefficients, *p*-values and R^2^ values were presented in Table 2. Figures S1–S9 also provide further details about the motility and its correlation with several factors. The linear regression models for the motility parameters (total motility, ProgA, ProgB) identified body weight, physical activity level and the FRAP antioxidant capacity index as the main significant predictors. Specifically, for total motility, body weight showed a statistically significant negative effect (*p* = 0.0167), whilst days of abstinence showed a marginally positive correlation (*p* = 0.049). The model explained 23% of the variance (R^2^ = 0.23). With regard to type A progressive mobility (ProgA), the FRAP index showed a strong positive effect (*p* = 0.001), whilst physical activity was also positively correlated (*p* = 0.0023). This model explained 24% of the total variance. In contrast, for progressive mobility type B (ProgB), the FRAP index showed a statistically significant negative effect (*p* = 0.011), whilst body weight also contributed negatively (*p* = 0.014). The proportion of explained variance was lower (R^2^ = 0.16). Overall, the mobility models indicate that body weight and physical activity are key determinants, whilst the effect of FRAP varies across mobility subtypes. However, the relatively limited explained variance (16–24%) suggests that mobility is correlated with additional, unaccounted-for factors. Overall, the mobility models indicate that body weight and physical activity are key determinants, whilst the effect of FRAP varies across mobility subtypes.

**Figure 1:**
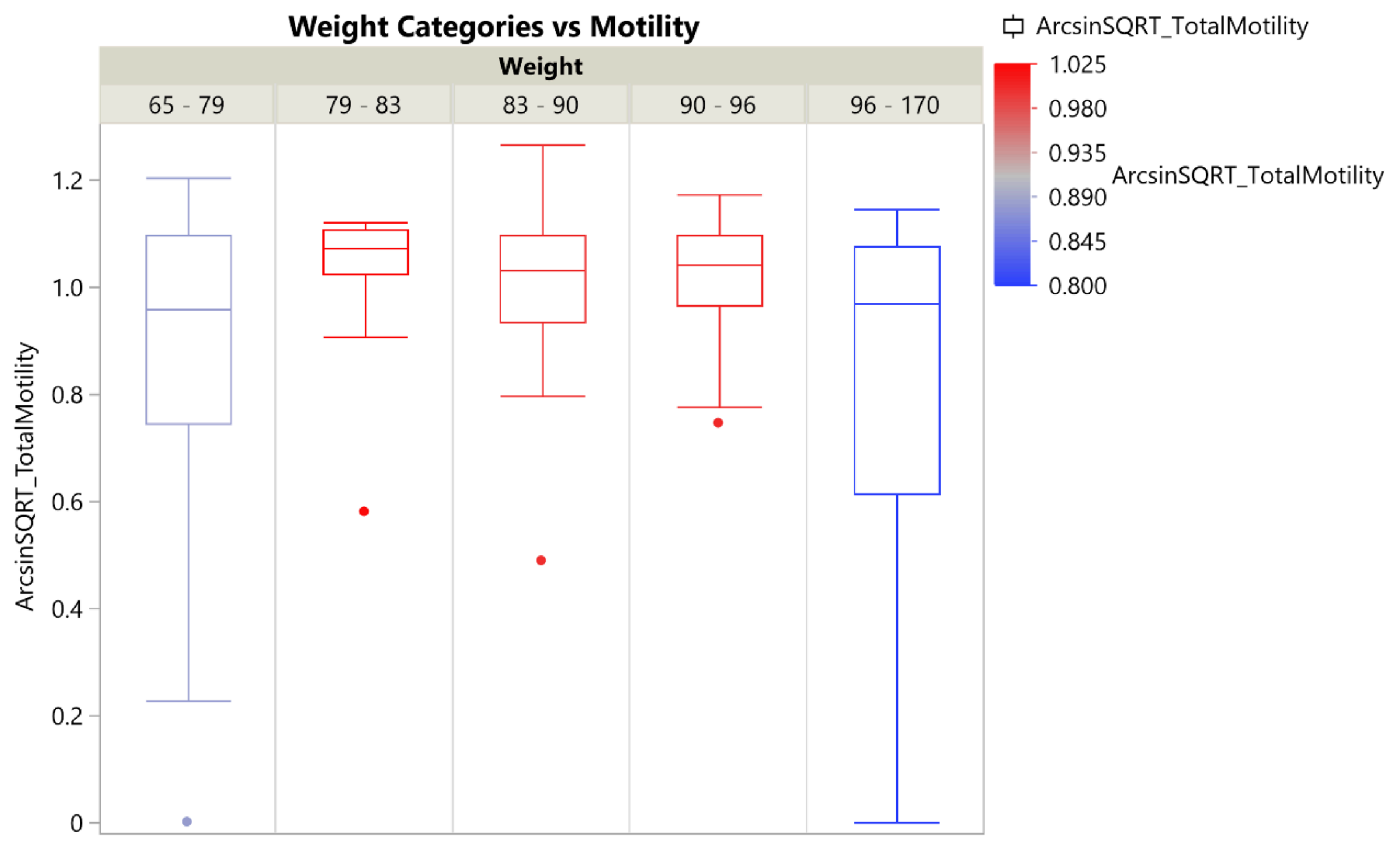
Distribution of total sperm motility (%) across weight categories. Boxplots illustrate the median, interquartile range (IQR), whiskers and individual outliers. Body weight categories were predefined for statistical comparison. Higher body weight categories were associated with lower total motility values.

**Figure 2:**
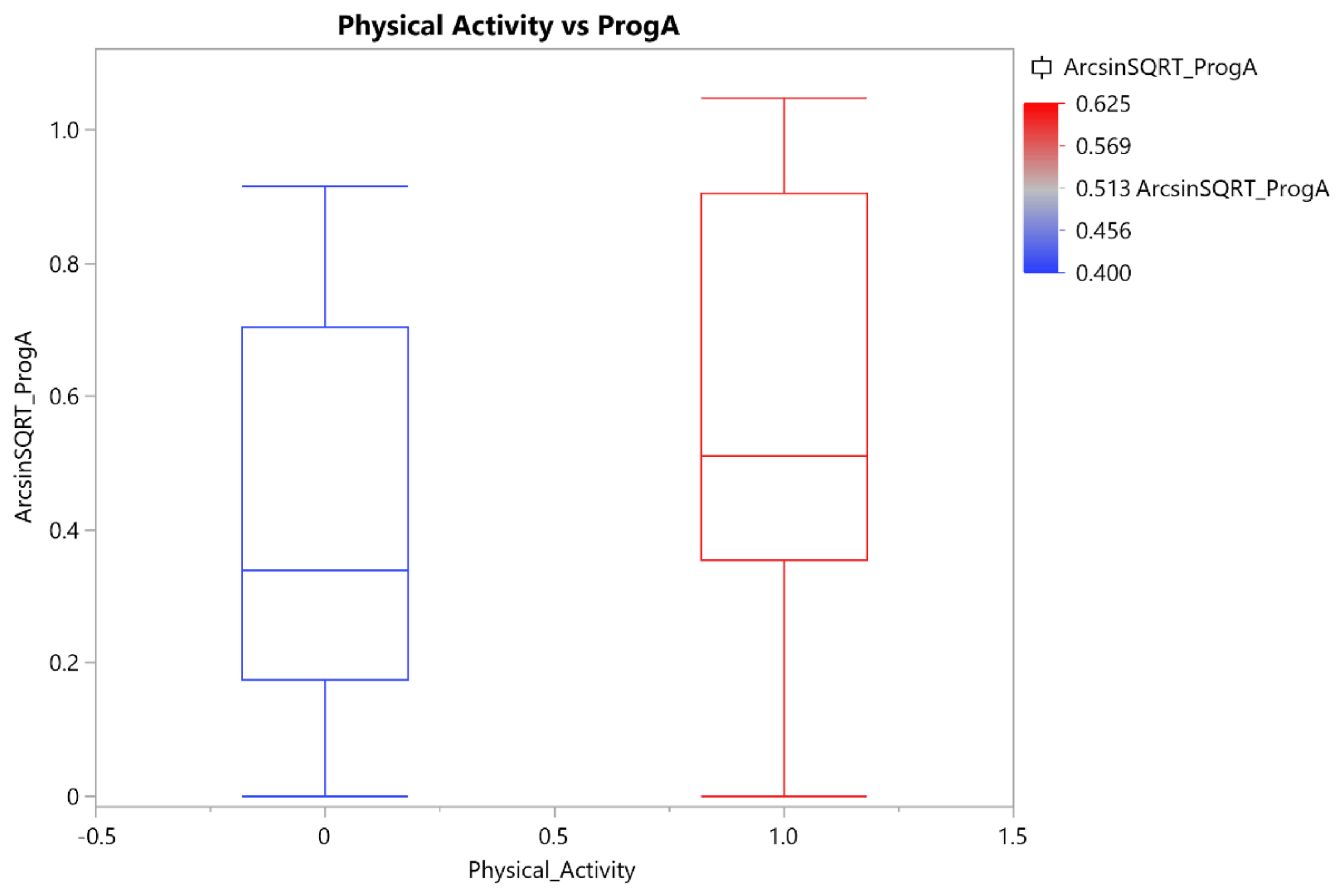
Progressive sperm motility (ProgA, %) according to physical activity levels. Boxplots present the median, interquartile range, whiskers and outliers. Participants classified as “minimally active” exhibited higher ProgA values compared with inactive individuals. Physical activity categories are displayed using JMP’s internal numeric coding: 0 = inactive, 1 = minimally active. Numeric spacing on the *x*-axis reflects software-generated category positioning.

It is known that mobility decreases with increasing body weight. Furthermore, the higher body weight categories exhibited lower mobility and concentration values, as shown in Figure 1, whereas higher physical activity also had positive results in sperm motility (Figure 2).

#### 3.3.2. Morphology

The analysis of morphology identified physical activity as the only statistically significant positive factor (*p* = 0.0048; R^2^ = 0.22), as shown in Table 3. The ORAC total index showed a marginal positive trend (*p* = 0.069), but did not meet the criterion for statistical significance. Physical activity emerged as the main positive predictor. This finding supports the hypothesis that physical activity is a favorable factor for sperm morphology, regardless of dietary antioxidant intake.

**Figure 3:**
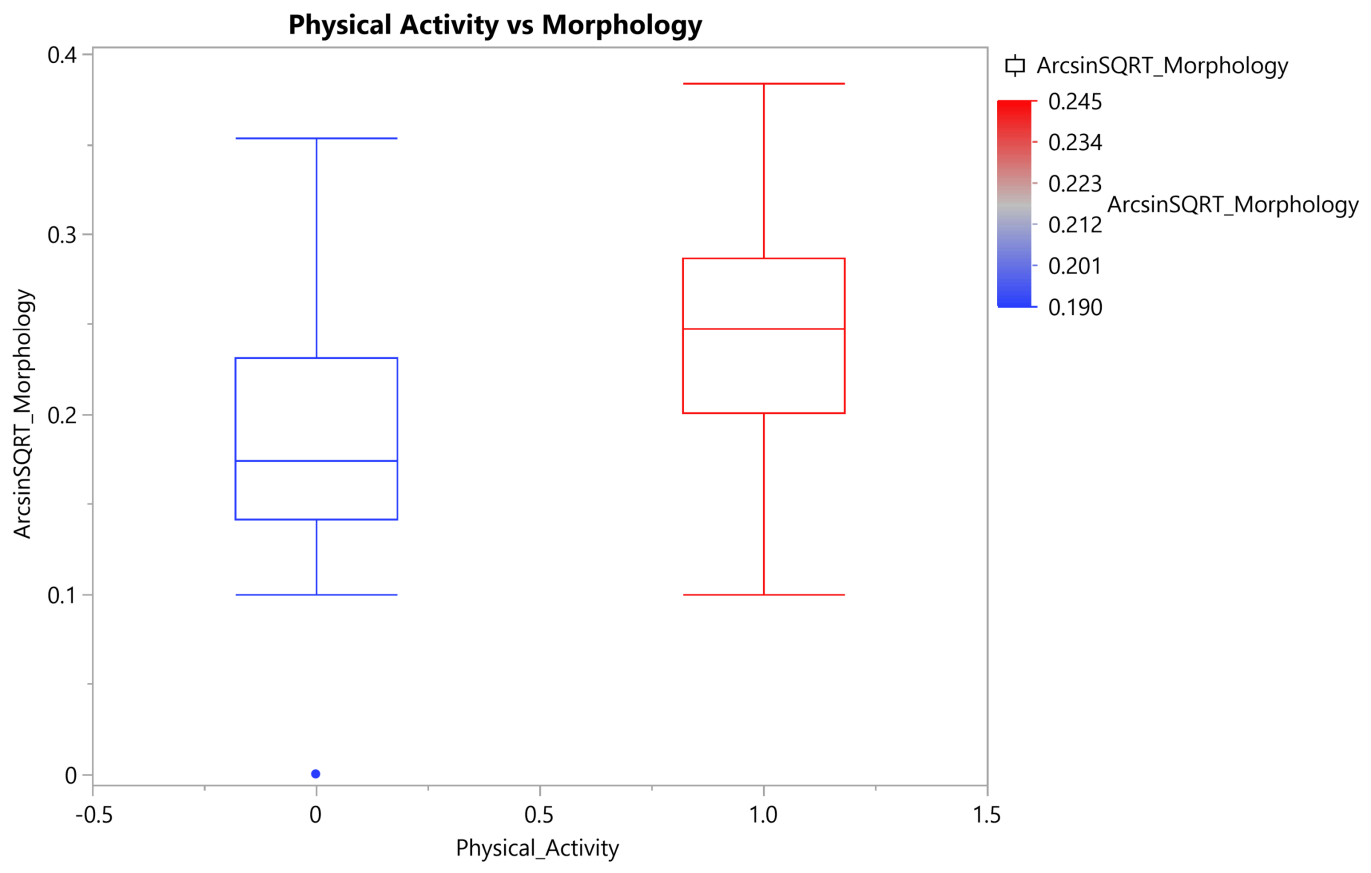
Percentage of morphologically normal spermatozoa according to physical activity levels. Boxplots display the median, interquartile range, whiskers and outliers. Increased physical activity was associated with higher percentages of normal sperm morphology. Physical activity categories are displayed using JMP’s internal numeric coding: 0 = inactive, 1 = minimally active. Numeric spacing on the *x*-axis reflects software-generated category positioning.

Higher levels of physical activity are associated with better morphology. Supplement use was associated with variations in vitality, whilst physical activity was associated with higher values for morphology and ProgA. Figures S10–S12 provide more details about the residual morphology of semen, whereas Figure 3 clearly showed the significance of increasing physical activity and sperm morphology.

#### 3.3.3. Sperm Concentration and Total Sperm Count

Regression models for sperm concentration and total sperm count identified smoking as the strongest negative predictor. In sperm concentration, smoking had a significant negative effect (*p* = 0.025), whilst body weight also had a negative effect on the variable (*p* = 0.029; R^2^ = 0.23). Figures S13–S15 provide further details about sperm concentration of individuals and especially how it was correlated with the smoking habit. For the total sperm count, smoking was the strongest negative factor (*p* = 0.0043), whilst days of abstinence had a marginally positive effect (*p* = 0.052; R^2^ = 0.25). Both smoking habit and increasing body weight significantly correlated with the individual’s sperm concentration and count (Figures S16–S18 also supported this evidence). These results (shown in Table 4) highlighted that smoking as the dominant negative factor for semen volume parameters, whilst body weight is mainly related with sperm concentration.

**Figure 4:**
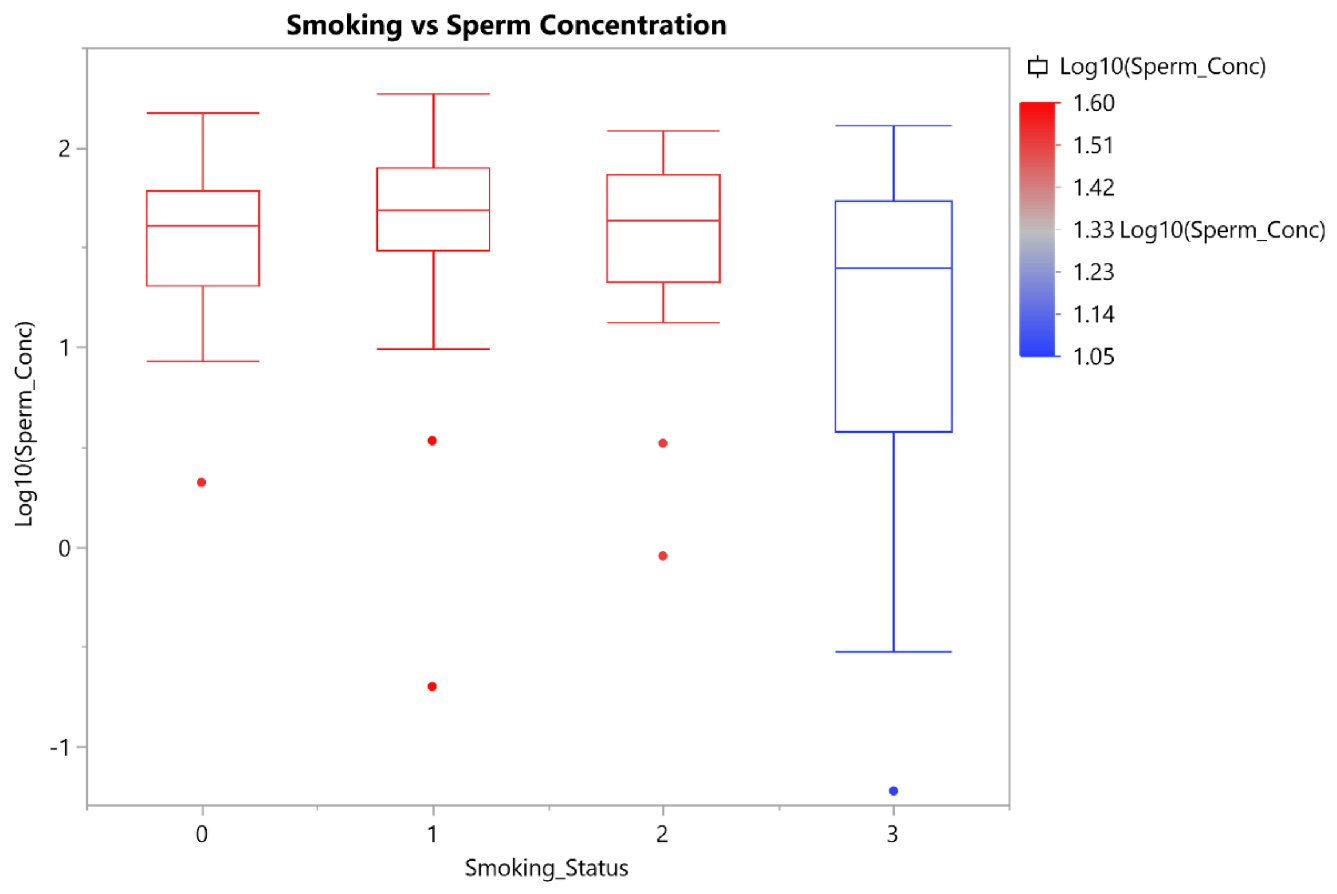
Distribution of sperm concentration across smoking categories. Smoking status was classified according to questionnaire responses and is displayed using numeric coding: 0 = non-smoker, 1 = light smoker, 2 = moderate smoker, 3 = heavy smoker. Boxplots present the median, interquartile range, whiskers and outliers. Higher smoking exposure was associated with lower sperm concentration.

**Figure 5:**
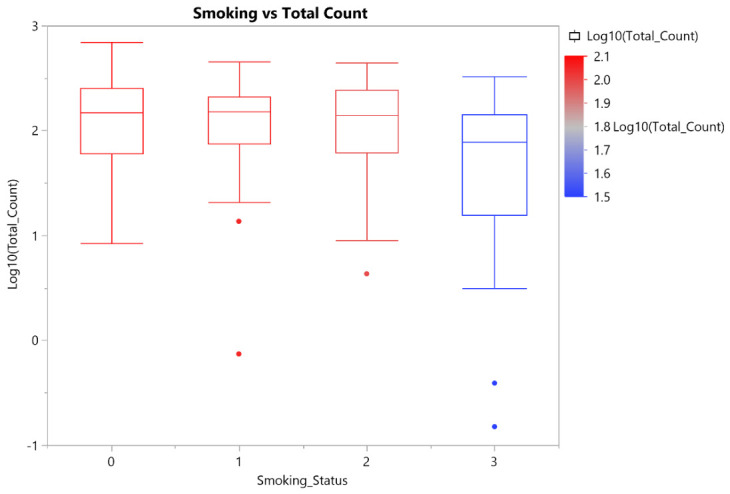
Distribution of total sperm count across smoking categories. Smoking status is displayed using numeric coding: 0 = non-smoker, 1 = light smoker, 2 = moderate smoker, 3 = heavy smoker. Boxplots illustrate the distribution of total sperm count among predefined smoking categories. Increased smoking exposure was associated with reduced total sperm count.

**Figure 6:**
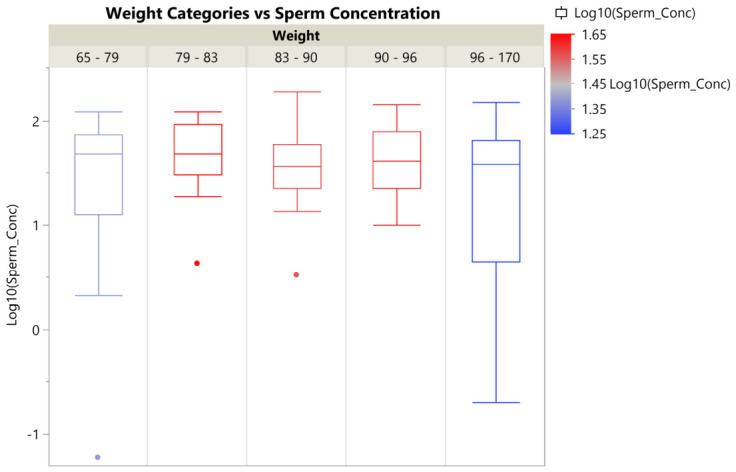
Sperm concentration across body weight categories. Boxplots show the distribution of sperm concentration according to predefined weight groups. Higher weight categories exhibited lower sperm concentration values.

The boxplots below (Figure 4, Figure 5 and Figure 6) indicate a clear and consistent downward trend in sperm quality parameters as the level of smoking increases. Smokers exhibit lower values for sperm concentration, total count, and motility, while greater dispersion and more outliers are observed in the higher smoking categories. This visual trend is fully consistent with the results of the Fit Models, where smoking emerged as the strongest negative factor for the parameters of quantity and motility. Smokers exhibited lower medians and greater variation in sperm concentration, with a clear downward trend as the level of smoking increases (Figure 4). A marked decrease in total count was also observed in the highest smoking categories, confirming the role of smoking as a strong negative factor (Figure 5). Finally, negative correlations were also observed with increasing weight and sperm concentration (Figure 6).

#### 3.3.4. Vitality

Sperm vitality was positively correlated with the use of dietary supplements (*p* = 0.0002), as well as with smoking (*p* = 0.033; R^2^ = 0.28), which had a negative effect. The results are shown in Table 5, whereas Figures S19–S21 indicated the marginal significance of physical activity and sperm quality. Physical activity and days of abstinence showed marginally positive correlations. Supplement use and smoking were significantly correlated with vitality. It is noteworthy that none of the antioxidant intake indices showed a statistically significant relationship with vitality.

**Figure 7:**
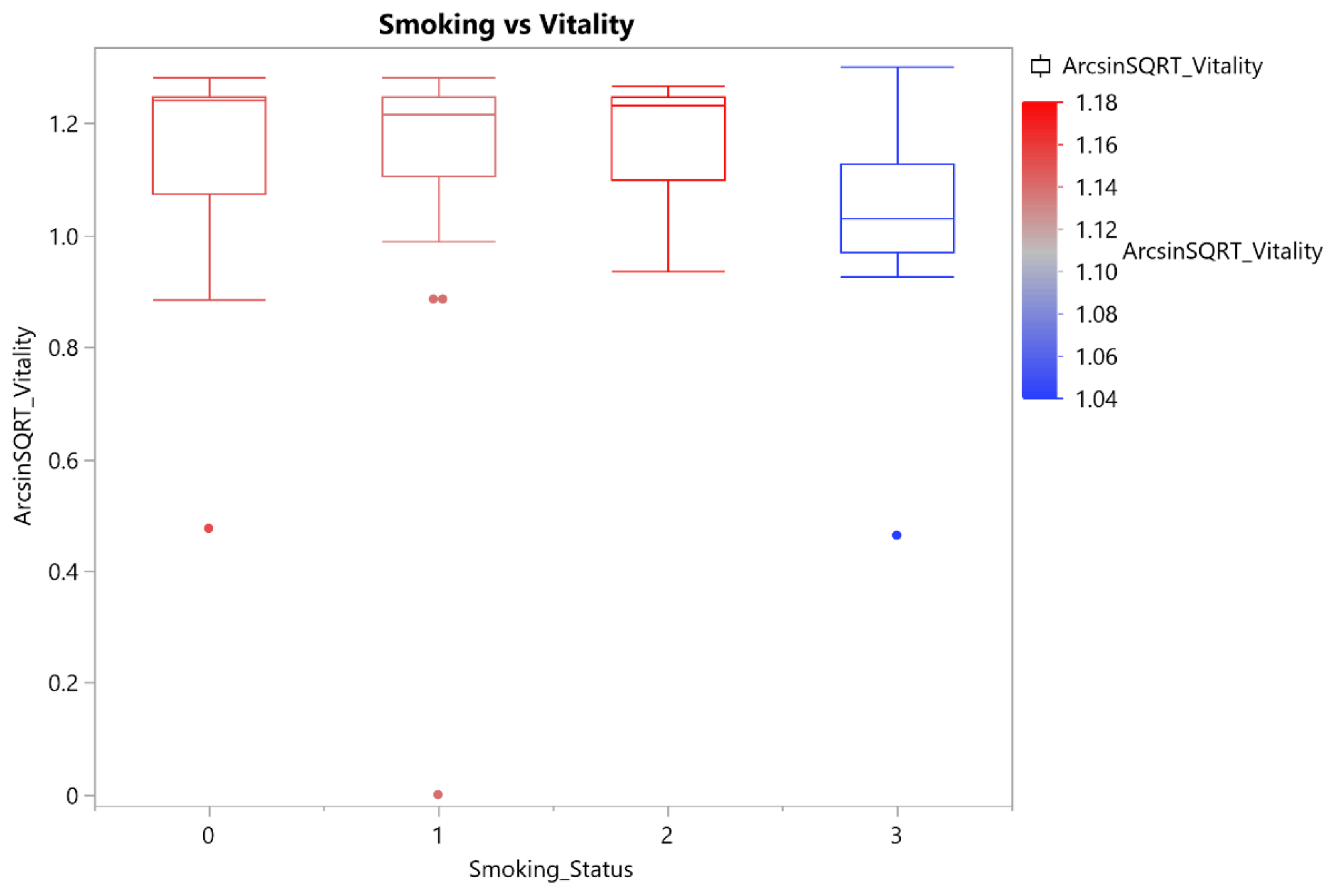
Sperm vitality (%) according to smoking categories. Smoking status is displayed using numeric coding: 0 = non-smoker, 1 = light smoker, 2 = moderate smoker, 3 = heavy smoker. Boxplots display the median, interquartile range, whiskers and outliers. Higher smoking exposure was associated with lower sperm vitality.

The analysis of standardized regression coefficients confirmed the relative strength of the factors. Body weight showed a consistently negative effect on multiple parameters (motility, ProgB, concentration and total count), with β values ranging from −0.17 to −0.25. Smoking emerged as the strongest negative factor for concentration (β = −0.29), total count (β = −0.36) and viability (β = −0.26) (vide infra). It was shown that lifestyle factors have a greater effect compared to antioxidant intake indices. The boxplot below (Figure 7) showed a clear and consistent downward trend in sperm quality parameters as the level of smoking increases. Smokers exhibit lower values for sperm concentration, total count, and motility, while greater dispersion and more outliers are observed in the higher smoking categories. This visual trend was fully consistent with the results of the Fit Models, where smoking emerged as the strongest negative factor for the parameters of quantity and motility.

The correlation analysis revealed several meaningful associations between lifestyle factors, semen antioxidant capacity, and sperm quality parameters. Smoking showed consistently negative correlations with nearly all semen parameters, most strongly with total sperm count (r = −0.365), concentration (r = −0.288), and vitality (r = −0.262) (vide infra). This finding aligns with the well-established detrimental effect of cigarette smoke constituents on spermatogenesis and sperm membrane integrity. In contrast, physical activity was positively correlated with total motility (r = 0.199), progressive A motility (r = 0.324), and morphology (r = 0.302), suggesting a protective role of regular exercise on sperm function, possibly through enhanced systemic oxidative balance.

Unexpectedly, the use of dietary supplements was negatively correlated with vitality (r = −0.393), the strongest inverse association observed. This finding may reflect reverse causation—individuals with pre-existing poor sperm quality may be more likely to use supplements—or could indicate excessive or inappropriate supplementation that disrupts redox homeostasis. Alternatively, unmeasured confounders such as type, dose, and duration of supplement use may explain this result. Overall, these correlations suggest that lifestyle modification (smoking cessation, increased physical activity) and targeted antioxidant support may be related with specific semen parameters differently. However, causal inference cannot be drawn from cross-sectional correlation data, and multivariate regression analyses adjusting for confounders are warranted to confirm independent associations.

**Figure 8:**
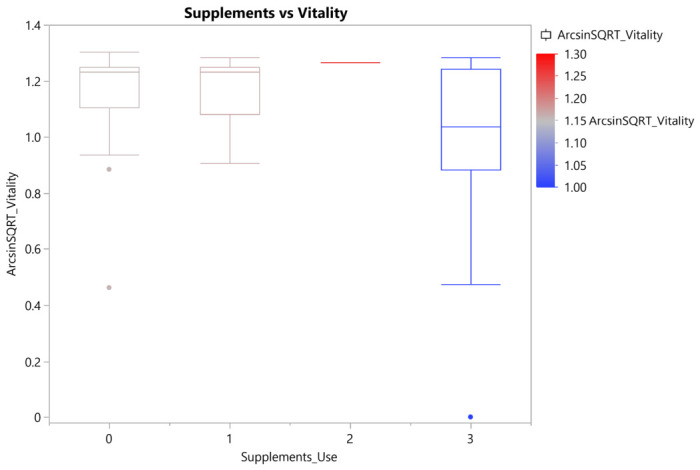
Distribution of sperm vitality (%) according to dietary supplements use. Boxplots compare vitality between participants reporting supplement use and those not using supplements. Differences are presented descriptively without implying causality.

Figure 8 showed a clear difference in vitality between the groups with and without supplement use. The group of supplement users exhibits a lower median and greater dispersion, suggesting that supplement use is associated with reduced vitality. This finding is fully consistent with the results of the Fit Model, where supplement use was the strongest negative factor (standardized β = −0.39), confirming that this variable has a significant and negative effect on sperm vitality.

In contrast, physical activity had a positive effect on morphology (β = 0.30), ProgA (β = 0.32), as well as other parameters of motility and viability, as shown in Table 6. The use of supplements was strongly associated with vitality, whilst the FRAP index showed a mixed effect (positive on ProgA and negative on ProgB). The ORAC and DPPH indices showed very small effect sizes (β < 0.15), with no substantial contribution to the models.

From a clinical perspective, conventional WHO semen parameters (concentration, motility, morphology, vitality) should be interpreted collectively rather than individually. Alterations in a single parameter do not necessarily indicate impaired fertility. The consistent associations observed between lifestyle factors and multiple semen parameters suggest that behavioral modifications may be related to overall reproductive health rather than isolated semen characteristics.

### 3.4. Correlation Analyses

#### 3.4.1. Principal Component Analysis (PCA)

**Figure 9:**
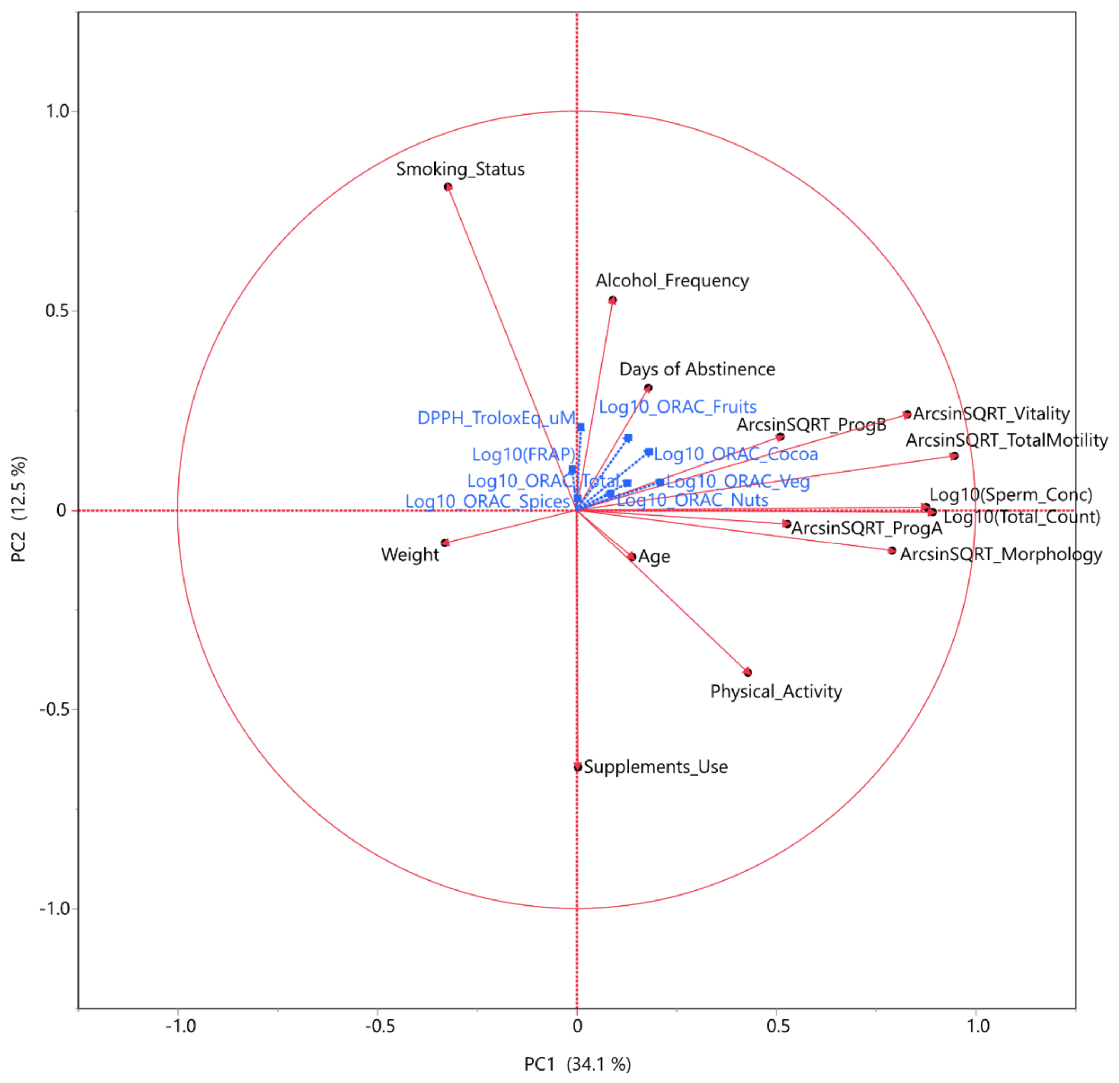
Principal component analysis (PCA) biplot illustrating relationships among semen quality parameters, demographic variables, lifestyle factors and antioxidant indices. Variables positioned close together indicate positive correlations, whereas variables located in opposite directions indicate negative correlations. Semen parameters clustered closely, while antioxidant indices formed a separate cluster with minimal contribution to the principal components.

We employed PCA to identify four principal axes of variation. The first component represented overall semen quality, whereas the second was associated mainly with lifestyle factors. Antioxidant markers did not show substantial associations with semen quality variables. The PCA biplot in Figure 9 illustrates the multidimensional structure of the dataset by projecting correlated variables onto the first two principal components. Variables pointing in similar directions indicate positive correlations, whereas variables pointing in opposite directions indicate inverse relationships.

The close clustering of semen quality parameters (motility, ProgA, ProgB, morphology, concentration, total count, vitality) suggests strong interrelationships among conventional WHO variables. In contrast, FRAP, DPPH and ORAC indices formed a separate cluster and were oriented orthogonally to the semen quality vectors, indicating minimal linear association with sperm parameters.

Lifestyle factors (smoking status, body weight, physical activity) and demographic variables formed distinct directions in the component space, reflecting the structure of the relationships identified in the regression models. Smoking and body weight aligned with lower semen quality vectors, whereas physical activity aligned with higher motility and morphology vectors.

This graphical distinction validated the regression model results, indicating that antioxidant indices did not significantly contribute to the heterogeneity in semen quality metrics. Overall, PCA confirmed that lifestyle variables exhibited stronger multivariate relationships with semen parameters than antioxidant indices.

#### 3.4.2. Multivariate Correlation Analysis

**Figure 10:**
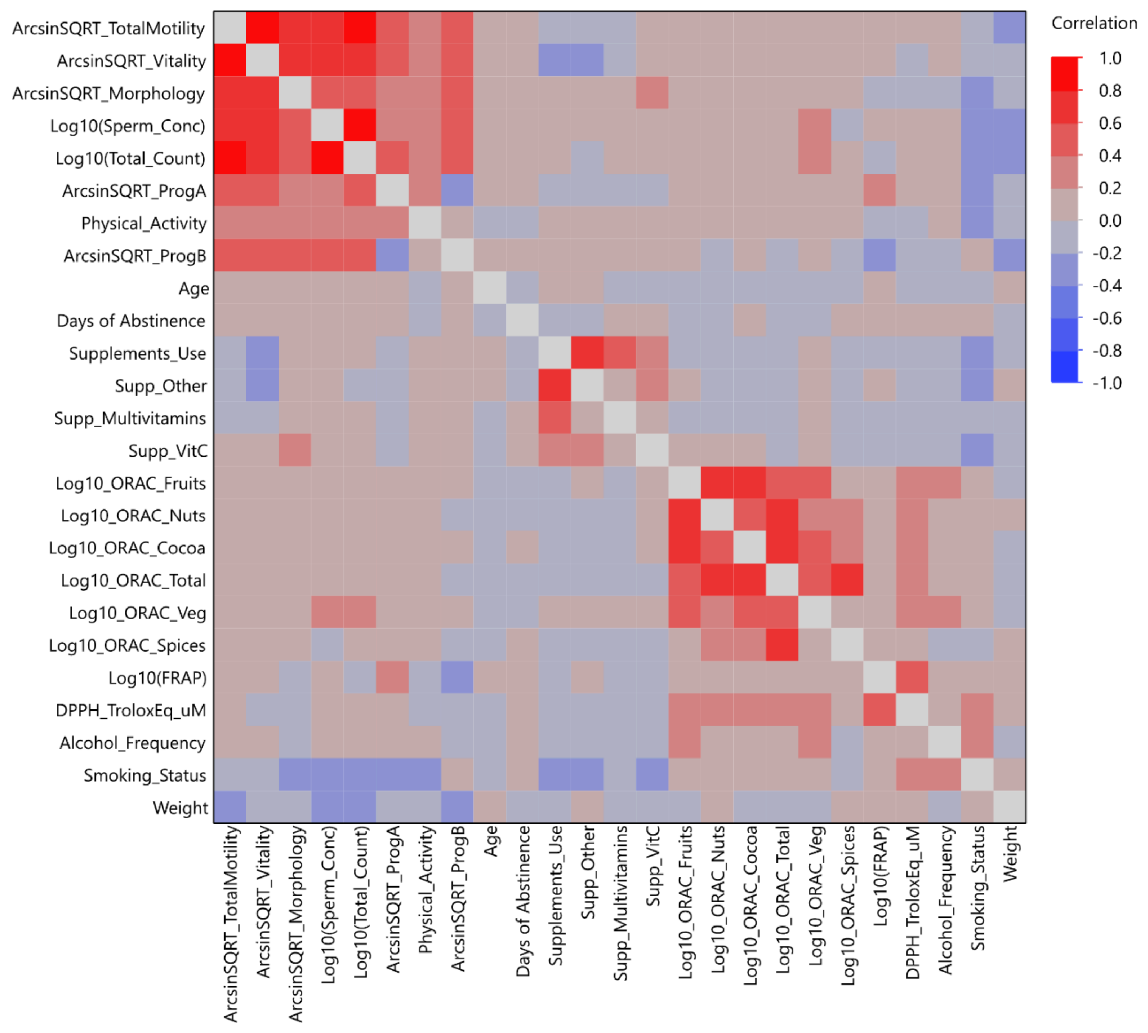
Correlation heatmap illustrating Pearson correlation coefficients among semen parameters, antioxidant indices and lifestyle variables. Red indicates strong positive correlations, blue indicates strong negative correlations and lighters colors represent weak or no correlation. Diagonal elements are displayed in neutral gray to distinguish self-correlations from standard correlation coefficients. The clustering pattern shows stronger relationships between lifestyle variables and semen quality than between antioxidant indices and semen parameters.

We also employed heatmap analysis to confirm the absence of strong correlations between antioxidant intake indices and semen quality (Figure 10). The correlation heatmap provides a visual representation of the Pearson correlation coefficients among all study variables. Strong positive correlations are represented by darker red colors, whereas strong negative correlations are represented by darker blue colors. Lighter colors indicate weak or no correlation.

Two distinct clusters were observed in the heatmap. Cluster 1 consisted of the WHO semen parameters, which exhibited moderate-to-strong positive inter-correlations (e.g., total motility with ProgA, r > 0.50), confirming the expected internal consistency of conventional semen quality metrics. Cluster 2 comprised the antioxidant intake indices (ORAC values), which showed strong inter-correlations (r > 0.70), indicating coherent dietary antioxidant consumption patterns.

Cross-correlations between the two clusters were consistently weak (r < 0.20) and appeared as pale-colored cells in the heatmap, indicating that dietary antioxidant intake, as quantified by the ORAC values, was not strongly associated with any WHO semen parameter in this sample.

Overall, PCA and heatmap analysis graphically supported the conclusions of the regression models. The clear separation between antioxidant markers and semen quality parameters in the multivariate space (Figure 9), together with the absence of strong cross-correlations (Figure 10), substantiated the conclusion that dietary antioxidant intake was not significantly associated with semen quality.

These findings also reinforced that modifiable lifestyle factors—particularly smoking and physical activity—exhibited stronger relationships with sperm quality than dietary antioxidant intake alone. The clustering of antioxidant indices, in contrast to semen parameters, suggests that although dietary antioxidant patterns were internally coherent, they did not correlate with sperm quality, likely due to the complex interplay of nutrient absorption, metabolic conversion and endogenous seminal plasma antioxidant defenses.

### 3.5. Overall Evaluation

In terms of overall impact on sperm quality, lifestyle and demographic characteristics were far more significant than dietary antioxidant intake (Table 7). Total motility, ProgB motility, vitality, and concentration were all negatively impacted by increasing body mass index, and smoking was consistently linked to reduced sperm concentration, total count, and vitality. Total motility, ProgA motility, morphology, and vitality were all positively correlated with exercise, suggesting that physical activity is a good predictor. Vitality was favorably correlated with supplement consumption, and overall motility, concentration, total count, and vitality all exhibited positive impacts of longer abstinence days, which likely reflected optimized sample collection. Interestingly, none of the dietary antioxidant consumption indices (DPPH, FRAP, ORAC) were shown to be statistically significantly associated with sperm quality measures. This trend indicates that changes in dietary antioxidant consumption alone may not have a direct and quantifiable effect on male reproductive health as much as other modifiable behaviors, such as quitting smoking, controlling weight, and engaging in regular physical exercise. Nevertheless, drawing any conclusions about cause and effect is not possible due to the cross-sectional design. While the absence of correlation with antioxidant tests does not necessarily disprove the existence of oxidative stress, it could be a result of limitations in the methods used to evaluate diet, the selection of plasma-based tests, or a lack of diversity in antioxidant consumption among the participants. These interactions can be further understood in future prospective studies if they include targeted antioxidant biomarkers and longer-term dietary data.

## 4. Discussion

The findings of this study demonstrate that lifestyle and demographic factors exert a substantially greater association with sperm quality than dietary antioxidant intake. Across all statistical analyses, antioxidant indices (ORAC, FRAP and DPPH) showed no meaningful associations with WHO semen parameters, whereas smoking, body weight and physical activity were consistently correlated with sperm concentration, motility and morphology.

More specifically, according to our study, smoking emerged as the strongest negative correlate of sperm quality, particularly for concentration, vitality and total sperm count. This agrees with previous studies linking smoking to increased oxidative stress, sperm DNA damage and impaired spermatogenesis [27]. Cigarette smoke contains toxic substances, including free radicals and heavy metals, which increase ROS production and oxidative stress in the male reproductive system. Smokers consistently exhibit higher sperm DNA fragmentation, reduced motility, and lower antioxidant capacity compared to non-smokers [28]. These findings highlight the importance of smoking cessation and avoidance of passive smoke exposure for male reproductive health [29].

Similarly, increased body weight was also negatively associated with motility and concentration, likely due to hormonal imbalance, chronic inflammation and oxidative stress. The recent meta-analysis by Wu & Zhang [30] identified body mass index (BMI) as a significant risk factor in male fertility assessment. Similarly, previous reviews reported that increasing body weight is associated with progressive deterioration in semen quantity and quality in a dose-dependent manner [31,32]. Obese men consistently exhibit lower sperm concentration, total sperm count, progressive motility, total motility, and normal morphology compared with normal-weight individuals. Even overweight status has been associated with impaired semen parameters, with more pronounced abnormalities observed in obesity [33]. These findings are consistent with the present study, in which increased body weight demonstrated significant negative associations with total motility, ProgB motility, sperm concentration, and total sperm count. Furthermore, analysis according to body weight categories revealed a bidirectional relationship between increased body weight and reduced semen quality and quantity, with higher weight categories exhibiting lower motility and concentration values.

Because height measurements were not collected, BMI could not be calculated. Therefore, body weight should be interpreted as a surrogate anthropometric indicator rather than a direct measure of adiposity. Future studies should incorporate BMI, waist circumference and body composition assessments to more accurately evaluate the relationship between adiposity and male reproductive function.

Importantly, body weight represents a modifiable risk factor. Long-term weight management strategies are considered essential for restoring oxidative balance and improving reproductive health outcomes [34,35]. Lifestyle interventions, including dietary modification, physical activity, and behavioral therapy, as well as medical approaches such as pharmacotherapy and bariatric procedures, have demonstrated potential benefits in improving hormonal and metabolic parameters during the preconception period [36].

Pharmacological agents used for weight loss, including metformin and glucagon-like peptide-1 (GLP-1) receptor agonists, appear to exert both beneficial and adverse effects on male reproductive health. Similarly, bariatric surgery may be associated with improvements in hormonal disturbances while producing variable effects on semen quality [37]. Current clinical guidelines therefore recommend screening for obesity-related complications, including infertility, in order to individualize weight-loss targets and therapeutic strategies [38]. Future research should employ more homogeneous cohorts and robust methodological designs to clarify the mechanisms linking BMI with male reproductive dysfunction and to strengthen the evidence regarding the effectiveness of lifestyle and medical interventions in improving fertility outcomes among obese men [39,40].

In contrast, physical activity showed beneficial associations with morphology and progressive motility, supporting evidence that moderate exercise is positively associated with reproductive and metabolic function. This is in accordance with the results of our study, as physical activity showed a statistically significant positive effect on progressive mobility type A (ProgA), sperm morphology and viability. It is important to note that the effects of physical activity appear to be dose-dependent, with moderate exercise providing the most favorable balance between antioxidant enhancement and oxidative stress reduction [29,41]. Both insufficient and excessive exercise may be adversely associated with male fertility. Excessive endurance training, including marathon or elite athletic training, has been associated with reduced testosterone levels, impaired sperm quality, increased oxidative stress, and infertility [7,42].

Regarding alcohol, the meta-analysis conducted by Nguyen-Thanh et al. [43] reported that alcohol intake was associated only with reduced semen volume, while no significant associations were identified regarding sperm concentration, motility, or morphology. Similarly, the review by Szabó et al. [44] concluded that occasional alcohol consumption does not appear to exert significant adverse reproductive effects. These findings are consistent with the present study, in which alcohol consumption demonstrated only a marginal association with semen volume and no significant correlation with other semen quality parameters.

Despite increasing scientific interest, inconsistencies in the definition and quantification of alcohol exposure across studies limit the comparability of findings. Consequently, further methodologically rigorous investigations are required to elucidate the effects of alcohol consumption on metabolic and reproductive health [45]. At present, reducing alcohol intake is generally recommended to optimize reproductive outcomes, while moderate consumption may not exert clinically significant adverse effects [46].

Regarding the use of dietary supplements, in our study supplement use was associated with vitality, possibly reflecting reverse causation, as men with poorer semen quality may be more likely to use supplements. Moreover, the effectiveness of supplements varies depending on dosage, antioxidant type and baseline antioxidant status. Despite these promising findings, the current evidence base remains limited. Although several studies indicate beneficial effects of dietary supplementation on semen parameters, robust evidence demonstrating improvements in pregnancy rates or live birth outcomes remains insufficient [47]. Many available studies are characterized by small sample sizes and methodological limitations. In our analysis, supplement use did not show positive associations with semen quality. Smoking habit remained the most significant lifestyle factor across multiple semen parameters.

Future research should therefore focus on identifying optimal antioxidant combinations, establishing appropriate dosages, and conducting large-scale, high-quality clinical trials to better define the efficacy of antioxidant supplementation in male infertility management [48].

Finally, another common finding of our study is that lifestyle and dietary modifications may partially reverse fertility impairment, highlighting the importance of early preventive interventions [49]. Although antioxidants appear beneficial, the literature also suggests that their effects depend on overall dietary balance rather than isolated supplementation, as synergistic interactions between nutrients are considered essential for reproductive health [50]. The absence of significant associations between antioxidant markers and semen quality suggests that dietary antioxidant intake alone may not accurately reflect the antioxidant environment of seminal plasma. Antioxidant defense was regulated not only by diet but also by endogenous enzymatic systems and metabolic factors. Additionally, methodological limitations such as dietary recall bias and inter-individual variability in antioxidant absorption may have reduced the ability to detect associations.

From a clinical perspective, conventional WHO semen parameters should be interpreted collectively rather than individually, as alterations in a single parameter do not necessarily indicate impaired fertility. The consistent associations observed between lifestyle factors and multiple semen parameters suggest that behavioral modifications may be related to overall reproductive health rather than isolated semen characteristics.

## 5. Limitations

Several limitations should be considered when interpreting the findings. Although the sample size was adequate for a semen study, variation in available WHO parameters may have reduced statistical precision. The lack of an a priori sample size computation was recognized. The sample size was dictated by participant availability throughout the research period, potentially constraining the generalizability of the results. In addition, semen quality naturally varies between ejaculations, introducing unavoidable biological variability.

Height was not documented in this study, and therefore body mass index (BMI) could not be computed. Body weight served as a surrogate anthropometric indicator; however, weight alone cannot differentiate between lean mass and fat mass. This limits the ability to determine whether the observed associations were related to excess adiposity or to other factors associated with increased body weight. Future research should incorporate objective anthropometric measurements such as height, waist circumference and body composition analysis to more accurately evaluate adiposity and its relationship with semen quality.

The food frequency questionnaire employed in this study was specifically designed for this experiment and did not measure absolute daily antioxidant intake; instead, it evaluated habitual consumption habits using category-based frequency responses. Semi-quantitative FFQs may introduce recall bias and provide limited precision in estimating nutrient intake. Likewise, physical activity was evaluated using only two classifications (‘inactive’ and ‘minimally active’), which offers restricted discriminating capacity. Future studies should consider validated standardized tools such as the International Physical Activity Questionnaire—Short Form (IPAQ-SF) or the Global Physical Activity Questionnaire (GPAQ), combined with quantitative dietary assessment methods such as 24 h recalls or food diaries, to facilitate more accurate and comparable analyses [51,52].

Another limitation concerns the ORAC index, which estimates dietary antioxidant intake from food databases rather than direct biological measurements. Consequently, ORAC values may not reflect actual antioxidant bioavailability or antioxidant activity within seminal plasma. The use of food frequency questionnaires may also introduce recall and measurement bias.

The cross-sectional design prevents causal interpretation of the observed associations. Residual confounding factors, including environmental exposures or hormonal disturbances, may also be associated with the results. Furthermore, post hoc power analysis indicated limited ability to detect very weak correlations between antioxidant indices and semen parameters.

Despite these limitations, the consistency of the findings across multiple statistical approaches strengthens the reliability of the conclusions and supports the major role of lifestyle factors in sperm quality.

## 6. Conclusions

The present study demonstrates that lifestyle factors are stronger associates of sperm quality than antioxidant intake indices. Smoking and increased body weight were associated with poorer semen parameters, whereas physical activity showed beneficial associations with motility and morphology. In contrast, ORAC, FRAP and DPPH indices did not show significant associations with the WHO semen parameters. These findings suggest that dietary antioxidant intake may not accurately represent antioxidant activity within seminal plasma, as antioxidant defense depends on multiple metabolic and endogenous mechanisms.

Lifestyle factors appear to be associated with oxidative stress, hormonal balance and the reproductive microenvironment, thereby exerting greater biological relevance for spermatogenesis than dietary antioxidant intake alone. Overall, the results emphasize the importance of lifestyle modification in the management of male fertility. Interventions targeting smoking cessation, weight reduction and increased physical activity are likely to have greater clinical relevance than dietary antioxidant intake alone.

Further studies with larger samples and more precise biomarkers are needed to clarify the role of antioxidants in male reproductive health.

## Figures and Tables

**Table 1:** Pearson correlations between antioxidant intake indices (ORAC, FRAP, DPPH) and sperm quality parameters (WHO parameters).

ORAC Category	Total Motility	ProgA	ProgB	Morphology	Sperm Concentration	Vitality
ORAC fruit	0.06	0.11	0.07	0.06	0.13	0.08
ORAC veg	0.08	0.16	0.1	0.09	0.2	0.1
ORAC nuts	0.04	0.09	0.06	0.08	0.05	0.06
ORAC cocoa	0.13	0.12	0.09	0.11	0.21	0.11
ORAC spices	0.05	0.12	0.06	0.03	0.11	0.09
ORAC total	0.09	0.13	0.07	0.11	0.19	0.12

**Table 2:** Linear regression models for motility parameters (total motility, ProgA, ProgB).

Response	Significant Predictors	Direction	*p*-Value	R^2^
Total motility	Weight	↓	0.0167	0.23
	Days of abstinence	↑	0.049	
ProgA	Log10 (FRAP)	↑	0.001	0.24
	Physical activity	↑	0.0023	
ProgB	Log10 (FRAP)	↓	0.011	0.16
	Weight	↓	0.014	

**Table 3:** Regression model for sperm morphology.

Response	Significant Predictors	Direction *	*p*-Value	R^2^
Morphology	Physical activity	↑	0.0048	0.22
	Log10_ORAC_Total	↑ (marginal)	0.069	

* ↑ indicates a positive association between the predictor and sperm morphology (i.e., higher predictor values correspond to higher morphology outcomes).

**Table 4:** Regression models for sperm concentration and total sperm count.

Response	Significant Predictors	Direction *	*p*-Value	R^2^
Log10 (Sperm_Concentration)	Smoking status	↓	0.025	0.23
	Weight	↓	0.029	
Log10 (Total_Count)	Smoking status	↓	0.0043	0.25
	Abstinence days	↑ (marginal)	0.052	

* ↑ indicates a positive association with the response variable; ↓ indicates a negative association.

**Table 5:** Regression model for sperm vitality.

Response	Significant Predictors	Direction *	*p*-Value	R^2^
Vitality	Supplement use	↓ (the more ↑ use → the more ↑ vitality)	0.0002	0.28
	Smoking status	↓	0.033	
	Physical activity	↑ (marginal)	0.062	
	Abstinence days	↑ (marginal)	0.064	

* ↑ indicates a positive association with sperm vitality; ↓ indicates a negative association.

**Table 6:** Standardized β-coefficients for sperm quality parameters.

Predictor	Total Motility	ProgA	ProgB	Morphology	Sperm Concentration	Total Count	Vitality
Age	0.156	0.057	0.128	0.132	0.162	0.173	0.101
Weight	−0.231	0.04	−0.248	−0.070	−0.215	−0.172	−0.100
Days of Abstinence	0.184	0.091	0.122	0.072	0.151	0.184	0.17
Smoking	−0.129	−0.105	−0.040	−0.138	−0.288	−0.365	−0.262
Physical Activity	0.199	0.324	−0.097	0.302	0.084	0.066	0.198
Use of Supplements	−0.052	−0.086	0.107	−0.138	−0.032	−0.069	−0.393
Log10 (FRAP)	0.037	0.371	−0.298	0	−0.043	−0.081	0.04
DPPH	0.052	−0.086	0.107	−0.138	0.091	0.077	−0.052
Log10_ORAC_Total	0.126	0.049	0.068	0.184	0.143	0.142	0.072

**Table 7:** Summary of lifestyle and demographic predictors that are correlated with WHO semen parameters.

WHO Parameter	Smoking	Weight	Physical Activity	Supplements	Alcohol	Abstinence
Total Motility	–	↓	↑ (Borderline)	–	–	↑
ProgA	–	–	↑	–	–	–
ProgB	–	↓	–	–	–	–
Morphology	–	–	↑	–	–	–
Sperm Concentration	↓	↓	–	–	–	↑ (Maximum)
Total count	↓	– (Threshold)	–	–	– (marginal)	↑ (Marginal)
Vitality	↓	–	↑ (Marginal)	↑	–	↑ (Borderline)

↑ indicates a positive association with the semen parameter; ↓ indicates a negative association; – denotes no significant association.

## Data Availability

The original contributions presented in the study are included in the article. Further inquiries can be directed to the corresponding author.

## Supplementary Materials

The following supporting information can be downloaded at: https://www.mdpi.com/article/10.3390/nu18152423/s1, Figure S1: Residuals vs. predicted for total motility; Figure S2: Studentized residuals for total motility; Figure S3: Leverage plot for weight (total motility model); Figure S4: Residual vs. predicted for ProgA; Figure S5: Studentized residuals for ProgA; Figure S6: Leverage plot for FRAP (ProgA Model); Figure S7: Residuals vs. predicted for ProgB; Figure S8: Studentized residuals for ProgB; Figure S9: Leverage plot for FRAP (ProgB Model); Figure S10: Residual vs. predicted for morphology; Figure S11: Studentized residuals for morphology; Figure S12: Leverage plot for physical activity (morphology model); Figure S13: Residual vs. predicted for sperm concentration; Figure S14: Studentized Residuals for sperm concentration; Figure S15: Leverage plot for smoking (sperm concentration model); Figure S16: Residual vs. predicted for total count; Figure S17: Studentized residuals for total count; Figure S18: Leverage plot for smoking (total count model); Figure S19: Residual vs. predicted for vitality; Figure S20: Studentized residuals for vitality; Figure S21: Leverage plot for physical activity (vitality model); Table S1: Volunteer code, age, weight, days of abstinence, and antioxidant capacity values (DPPH and FRAP) of the samples, with their statistics; Table S2: Questionnaire on the consumption of antioxidant foods; Table S3: Health and lifestyle questionnaire.

## Abbreviations

The following abbreviations are used in this manuscript:

BMI	Body Mass Index
DPPH	2,2-Diphenyl-1-Picrylhydrazyl
FFQ	Food Frequency Questionnaire
FRAP	Ferric-Reducing Antioxidant Capacity
GLP-1	Glucagon-like Peptide-1
GPAQ	Global Physical Activity Questionnaire
IPAQ	International Physical Activity Questionnaire—Short Form
ORAC	Oxygen Radical Absorbance Capacity
ProgA	Progressive Motility Type A
ProgB	Progressive Motility Type B
ROS	Reactive Oxygen Species

## Appendix A

**Table A1:** Linear equation details for each antioxidant assay.

Assay	Linear Equation	Range	R^2^	LOD	LOQ
FRAP (μmol Trolox eq/L)	*y* = 0.002*x* + 0.01	50–500	0.9993	17.82	53.99
DPPH (μmol Trolox eq/L)	*y* = 0.056*x* + 2.48	50–500	0.9989	26.48	80.25
