# Supplementary material for: Antioxidant Capacity of Seminal Plasma and Its Association with Semen Quality and Dietary Antioxidant Intake
Source: Nutrients. 2026 Jul 24;18(15):2423. doi: 10.3390/nu18152423 (PMC13468480; doi:10.3390/nu18152423)
Supplement: Supplementary file 1 [file nutrients-18-02423-s001.zip › nutrients-4392393-supplementary.pdf]

nutrients

MDPI

Supplementary Materials

Antioxidant Capacity of Seminal Plasma and Its Association with Semen Quality and Dietary Antioxidant Intake

Georgia Lagodonti [1], Dimitrios Kalompatsios [2], Panagiotis Varagiannis [2], Vassilis Athanasiadis [2] and Stavros I. Lalas [2,*]

[1] Department of Chemical Engineering, University of Western Macedonia, 50100 Kozani, Greece; georgia_lag@hotmail.com
[2] Department of Food Science & Nutrition, University of Thessaly, Terma N. Temponera Street, 43100 Karditsa, Greece; dkalompatsios@uth.gr (D.K.); pvaragiannis@uth.gr (P.V.); vaathanasiadis@uth.gr (V.A.)
* Correspondence: slalas@uth.gr (S.I.L); Tel.: +30-24410-64783

Total Motility

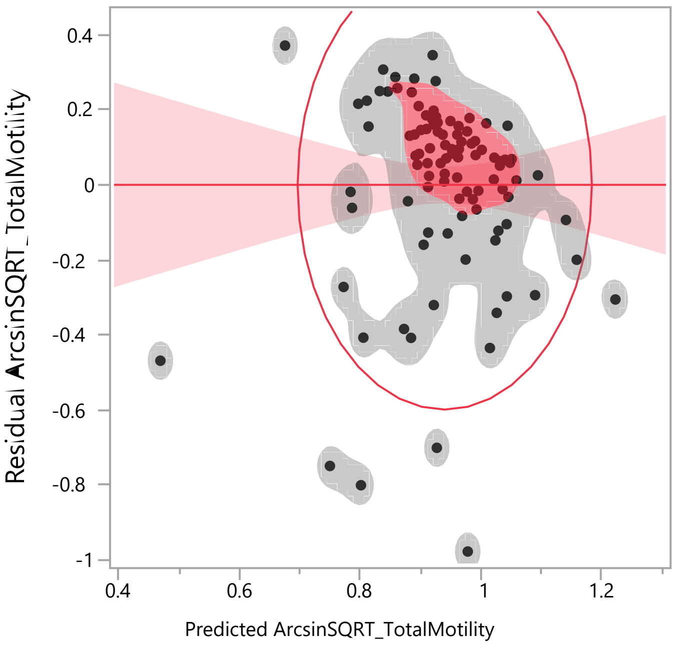

Figure S1. Residuals vs Predicted for Total Motility.

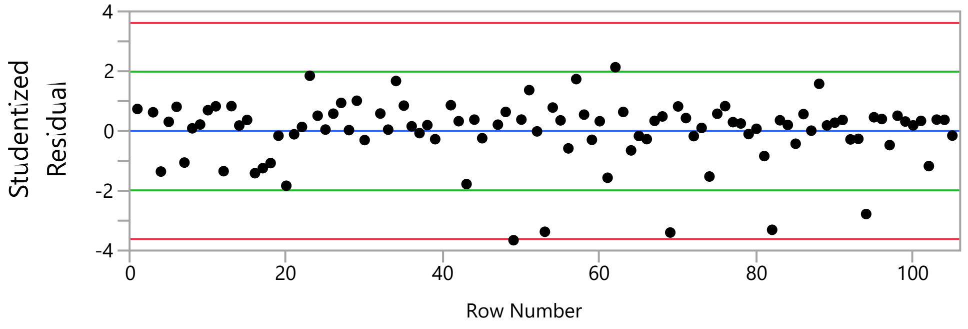

Figure S2. Studentized Residuals for Total Motility.

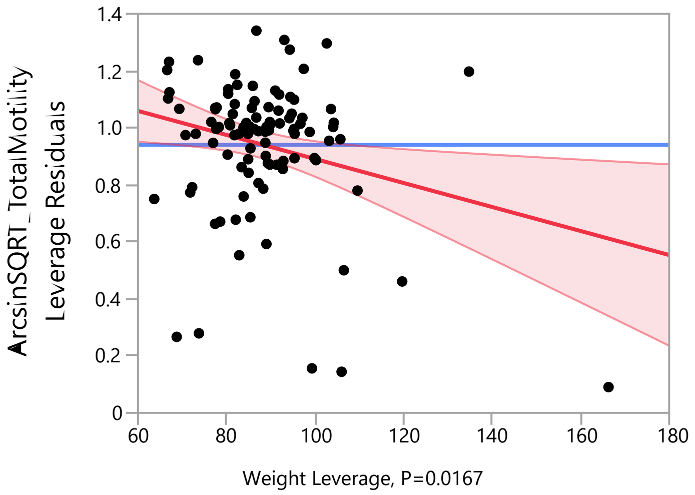

Figure S3. Leverage Plot for Weight (Total Motility Model).

https://doi.org/10.3390/xxxxx

ProgA

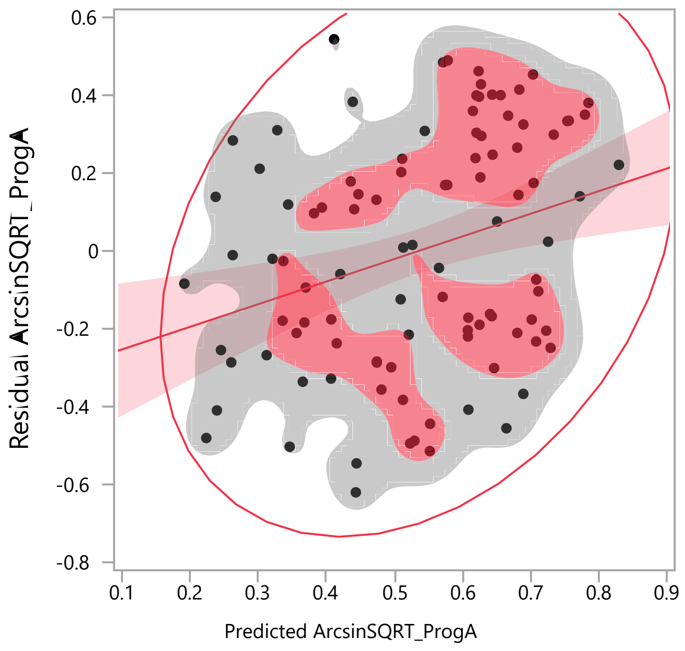

Figure S4. Residual vs Predicted for ProgA.

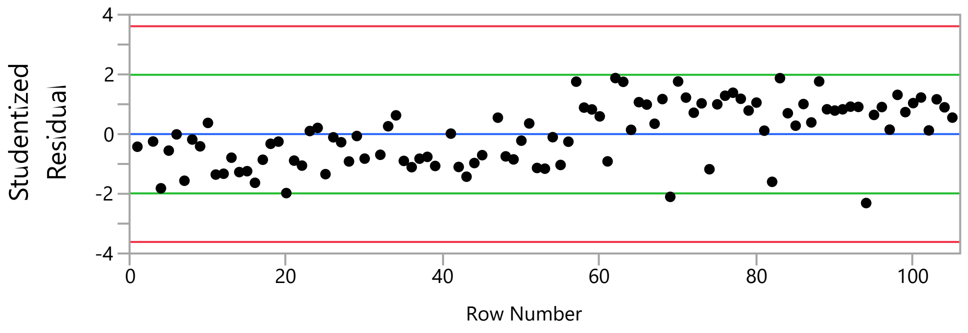

Figure S5. Studentized Residuals for ProgA.

https://doi.org/10.3390/xxxxx

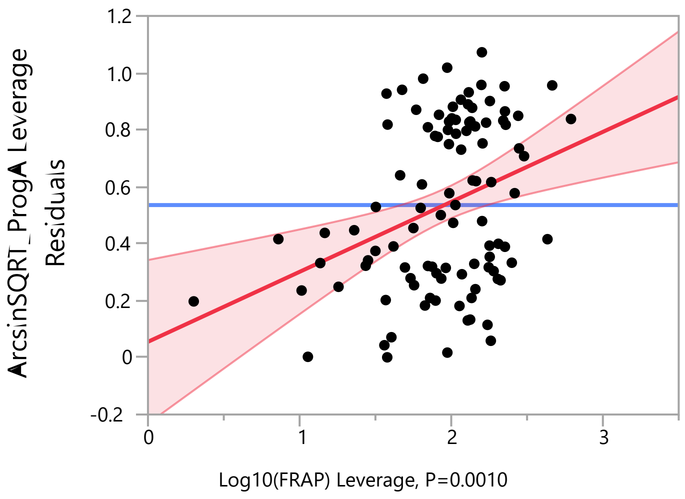

Figure S6. Leverage Plot for FRAP (ProgA Model).

ProgB

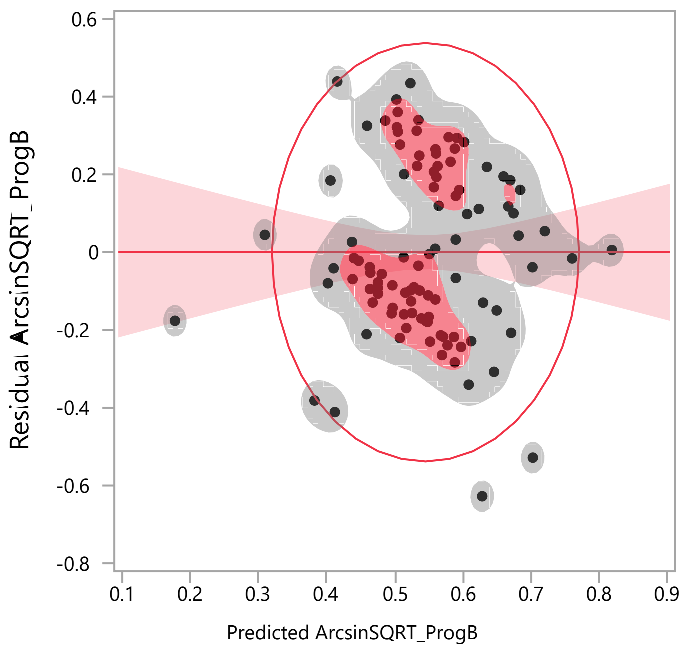

Figure S7. Residuals vs Predicted for ProgB.

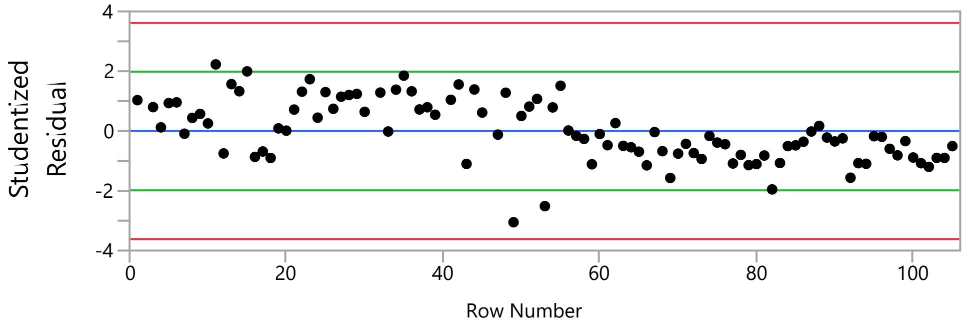

Figure S8. Studentized Residuals for ProgB.

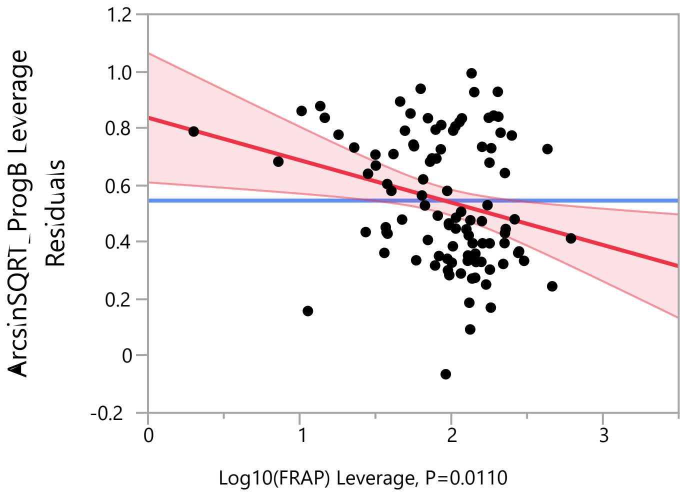

Figure S9. Leverage Plot for FRAP (ProgB Model).

Morphology

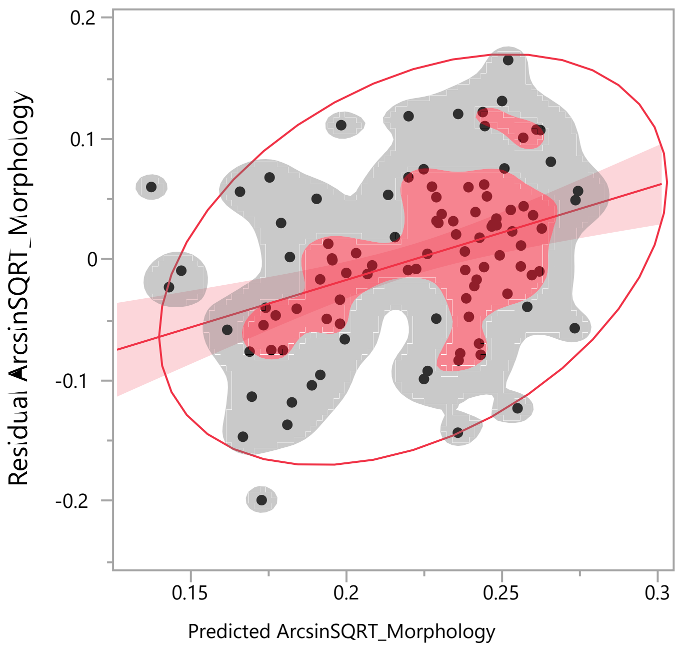

Figure S10. Residual vs Predicted for Morphology.

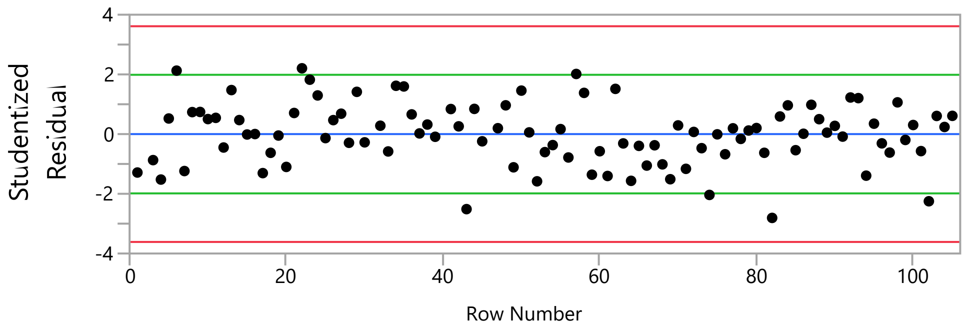

Figure S11. Studentized Residuals for Morphology.

https://doi.org/10.3390/xxxxx

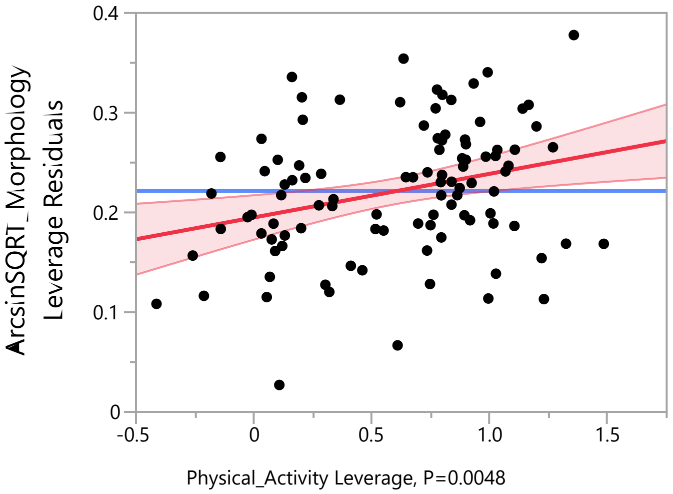

Figure S12. Leverage Plot for Physical Activity (Morphology Model).

Sperm Concentration

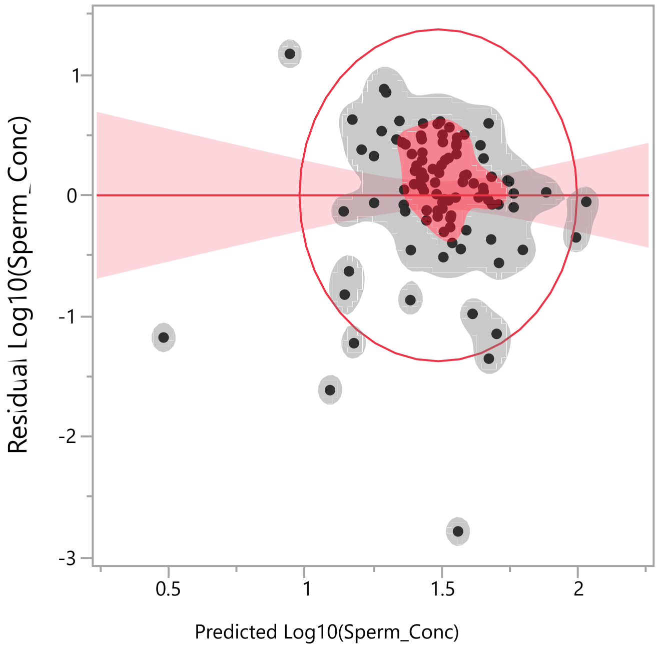

Figure S13. Residual vs Predicted for Sperm Concentration.

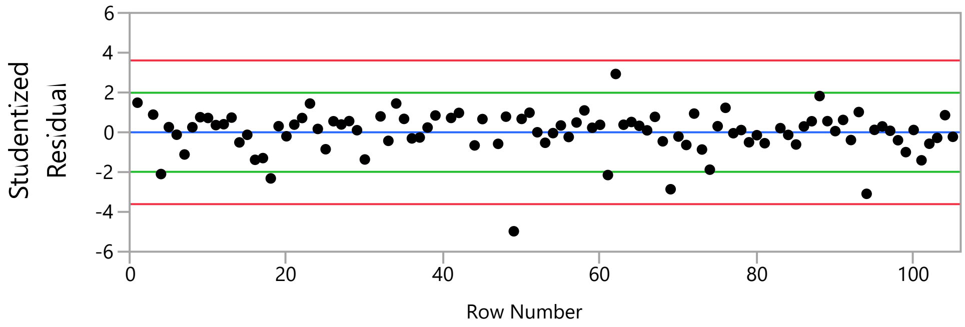

Figure S14. Studentized Residuals for Sperm Concentration.

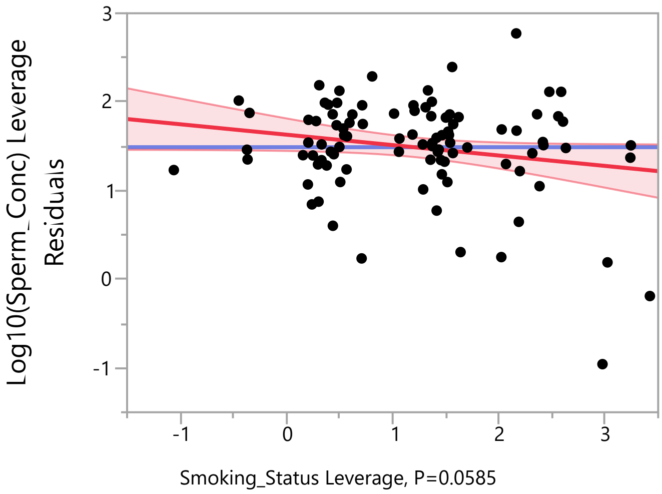

Figure S15. Leverage Plot for Smoking (Sperm Concentration Model).

Total Count

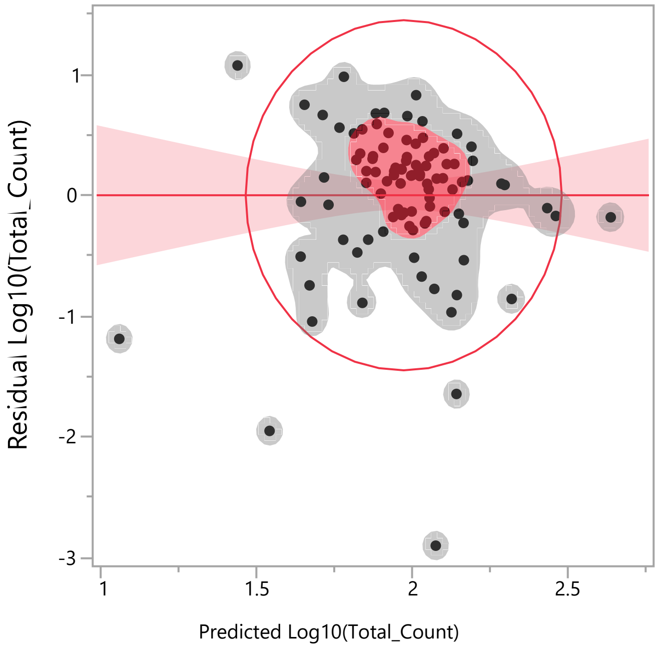

Figure S16. Residual vs Predicted for Total Count.

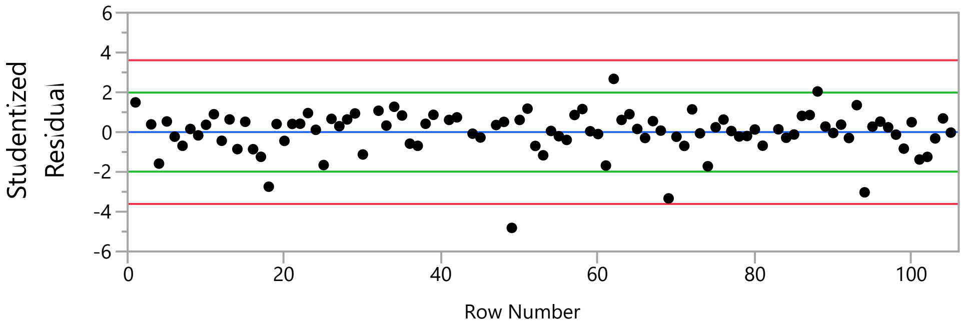

Figure S17. Studentized Residuals for Total Count.

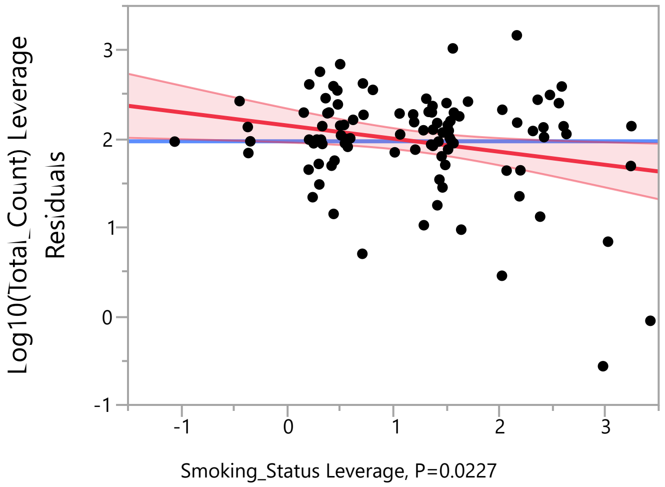

Figure S18. Leverage Plot for Smoking (Total Count Model).

Vitality

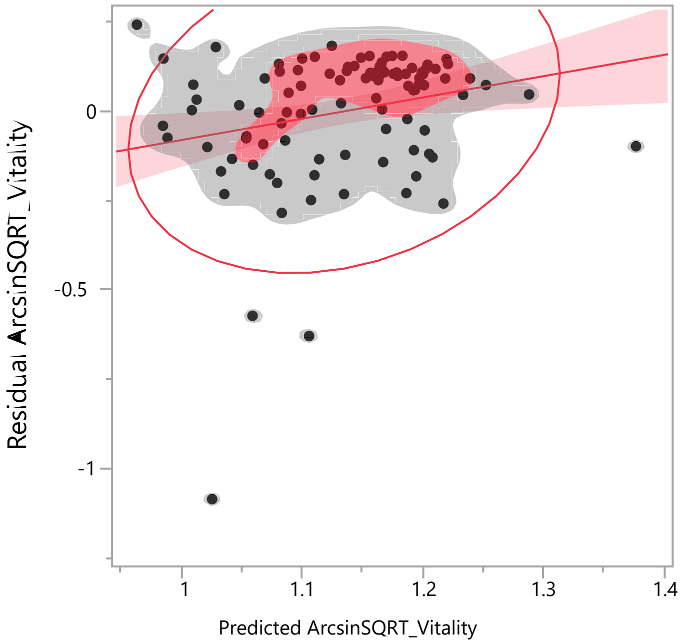

Figure S19. Residual vs Predicted for Vitality.

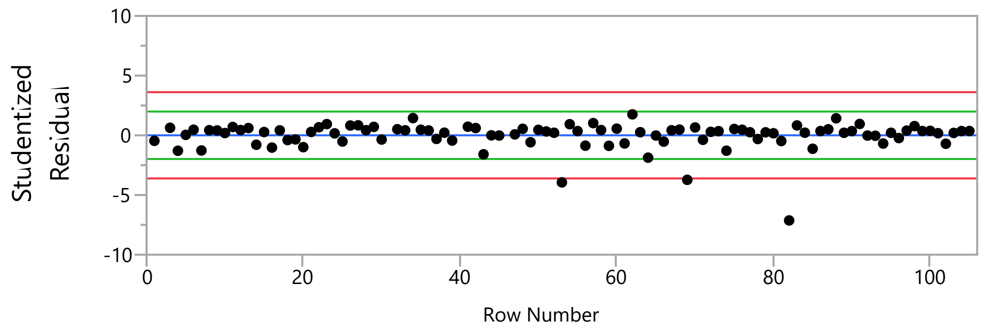

Figure S20. Studentized Residuals for Vitality.

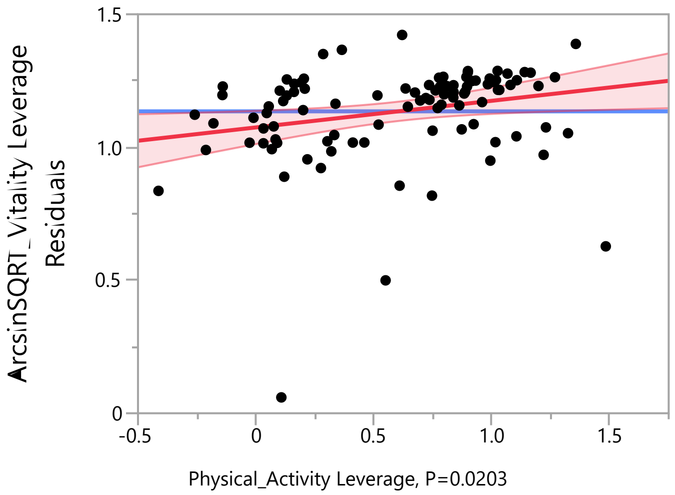

Figure S21. Leverage Plot for Physical Activity (Vitality Model).

Table S1. Volunteer code, age, weight, days of abstinence, and antioxidant capacity values (DPPH and FRAP) of the samples, with their statistics.

Patient_ID	Age	Weight	Days of Abstinence	DPPH (μmol Trolox eq/L)	FRAP (μmol Trolox eq/L)
2168	39	71	3	619.9	118.3
2171	28	72	4	748.1	-
2200	32	82	3	767.2	8.3
2225	40	75	3	1258.2	326.3
2247	36	65	3	934.6	51.0
2249	34	90	3	743.6	12.3
2251	59	75	3	1552.3	249.5
2252	43	77	4	418.6	17.9
2281	36	81	3	789.0	16.0
2282	38	81	3	705.5	33.3
2293	32	105	3	1161.3	250.0
2296	28	73	2	619.9	93.2
2297	33	85	3	796.1	189.7
2301	47	89	3	911.8	338.7
2310	34	75	3	1,212.3	364.0
2316	27	79	4	1199.8	267.9
2317	38	86	2	788.4	37.0
2319	21	70	4	1224.8	53.6
2320	41	95	3	768.1	31.7
2321	31	78	7	949.3	64.9
2322	50	80	3	534.9	192.4
2323	48	80	3	785.9	559.6
2324	47	110	3	397.2	45.6
2329	37	80	3	192.0	7.4
2330	43	75	3	816.4	113.5
2425	41	92	3	556.7	46.1
2426	33	85	4	702.5	104.2
2427	23	78	3	948.7	79.5
2476	44	80	3	499.3	73.2
2477	32	112	3	1043.1	161.2
2479	39	80	3	558.0	-
2480	39	98	5	348.3	53.7
2520	42	82	3	648.2	1.6
2522	34	96	3	728.3	43.7
2523	33	89	3	521.9	78.8
2799	37	81	3	706.8	202.9
2802	38	98	3	816.4	77.2
2809	39	68	3	664.4	123.5
2810	41	89	3	742.8	67.3
2815	31	88	3	621.0	-
2818	36	71	3	542.4	122.5
2821	34	94	3	804.3	178.9
2823	29	78	3	590.8	22.1
2825	32	82	4	403.6	58.2
2826	37	85	4	1157.7	154.0
2827	40	82	4	338.5	-
2829	33	85	3	466.7	3.7
2831	52	105	3	571.6	43.2

https://doi.org/10.3390/xxxxx

2833	42	75	3	28.5	24.6
2834	42	89	3	650.6	32.6
2837	51	98	3	457.2	11.3
2838	43	98	3	623.2	62.4
2860	37	72	2	621.1	6.6
2864	39	85	3	569.8	67.5
2865	33	75	3	842.2	172.7
3024	44	86	3	614.0	195.9
3025	31	86	3	1011.4	172.8
3027	35	101	3	787.6	84.6
3029	47	79	3	997.9	184.6
3031	34	102	3	411.7	60.4
3033	38	110	3	586.5	163.6
3035	35	140	3	1455.5	273.8
3036	32	80	3	665.1	38.2
3037	43	85	3	574.0	103.1
3038	43	74	3	624.5	60.0
3039	56	90	5	584.9	110.7
3040	37	82	3	725.0	523.1
3045	34	81	3	802.2	94.1
3047	35	107	2	807.3	143.5
3048	34	90	2	491.5	17.7
3049	22	75	3	656.3	164.3
3050	36	74	3	721.9	161.3
3051	40	83	3	483.5	59.1
3059	41	120	3	746.0	73.0
3062	42	95	3	677.8	87.4
3069	27	90	3	699.0	95.0
3073	42	89	3	440.2	33.9
3075	26	92	5	693.2	144.3
3077	41	83	4	842.9	213.4
3078	44	80	3	472.5	84.1
3080	34	83	3	882.1	156.6
3083	31	105	3	719.9	138.8
3084	35	89	3	3093.2	2171.4
3086	30	83	3	767.1	242.1
3087	34	90	4	828.4	376.1
3088	36	90	3	554.2	62.7
3090	44	98	3	1308.7	159.1
3092	26	101	3	765.9	88.9
3094	48	90	3	517.1	96.0
3104	35	88	3	763.0	107.2
3105	38	95	3	758.3	285.9
3106	34	80	5	869.4	177.5
3107	41	95	3	805.6	125.8
3108	36	170	3	573.7	116.8
3114	40	95	3	794.6	233.8
3117	34	110	3	617.5	112.3
3126	43	85	3	1004.5	432.0
3133	42	98	3	567.1	156.0
3137	42	90	3	1083.4	166.4
3139	44	78	3	815.4	175.3

https://doi.org/10.3390/xxxxx

3160	45	76	3	936.4	154.6
3162	51	84	4	774.8	107.0
3167	37	95	4	990.4	120.9
3168	45	93	3	442.8	260.7
3169	37	84	3	653.5	121.9
Mean	38	88	3	754	151
Std Dev	7	14	1	338	230
Std Err Mean	1	1	0	33	23
Upper 95% Mean	39	91	3	819	196
Lower 95% Mean	36	85	3	688	105
N	105	105	105	105	101
CV	18.1	16.3	20.8	44.9	152.6

https://doi.org/10.3390/xxxxx

Questionnaire

Table S2. Questionnaire on the Consumption of Antioxidant Foods.

Please indicate how often you consume the following foods.

One portion refers to the standard amount listed in the table.

You can select: rarely (1), 1–2 times a week (2), daily (3).

You can write '0' if you do not eat that particular food at all.

**Food**	**Category**	**Serving (g/mL)**	**ORAC per serving (μmol TE)**	**Frequency of consumption (to be completed by the participant)**
Clove (dried)	Spices	1	2903	
Cinnamon (dried)	Spices	1	1314	
Oregano (dried)	Spices	1	1753	
Dark chocolate (>70% cocoa)	Sweets/Snacks	20	4163	
Cocoa powder (unsweetened)	Sweets/Snacks	5	2783	
Nuts	Nuts	30	4062	
Almonds	Nuts	30	1336	
Grapes (red)	Fruit	100	1837	
Pomegranate (fresh)	Fruit	100	2860	
Blueberries (cultivated)	Fruit	100	9019	
Strawberries (fresh)	Fruit	100	5938	
Blackberries (fresh)	Fruit	100	7701	
Spinach (raw)	Vegetables	100	1513	
Broccoli (boiled)	Vegetables	100	1510	
Tomato (fresh)	Vegetables	100	546	
Olive oil (extra virgin)	Fats/Oils	15	56	
Red wine	Beverages	150	5810	
Green tea (infusions)	Beverages	200	2506	
Mountain tea (infusion)	Beverages	200		
Sage	Beverages			
Greek coffee (infusion)	Beverages	100	2500	
Black pepper (dried)	Spices	1	1310	
Turmeric (dried)	Spices	1	1271	
Dried cranberries	Fruit	40	3834	
Raisins	Fruit	40		
Carrots (raw)	Vegetables	100	666	
Beetroot (boiled)	Vegetables	100	1776	
Apple (with skin)	Fruit	150	4242	
Orange	Fruit	150	2728	
Weight:				
Participant's signature:				

https://doi.org/10.3390/xxxxx

Table S3. Health and Lifestyle Questionnaire

Instructions for completion

This questionnaire has been designed to assess lifestyle factors that may affect health and, in particular, reproductive function.

Please answer all questions honestly.
The data is anonymous and will be used exclusively for research purposes.

Demographic details

1. Participant ID (full name or email):

………………………………………………………………………

Lifestyle factors

2. Alcohol consumption

How often do you consume alcoholic drinks?
(100 ml = 12 g of ethanol)

☐ 0 – Never
☐ 1 – Rarely
☐ 2 – 1–2 times a week
☐ 3 – Daily
☐ 4 – 2 drinks daily

---

3. Smoking status

What is your current smoking status?

☐ 0 – Never smoked
☐ 1 – Former smoker
☐ 2 – Current smoker (<10 cigarettes/day)
☐ 3 – Current smoker (≥10 cigarettes/day)

---

4. Physical activity

How physically active are you (MET-min/week)?

☐ 0 – Inactive

☐ 1 – Minimally active

---

5. Use of dietary supplements

Do you use dietary supplements?

☐ 0 – Never

☐ 1 – Monthly or a few times a year

☐ 2 – Weekly

☐ 3 – Daily

---

6. Type of dietary supplements

What kind of supplements do you use?

(You may select more than one)

☐ Multivitamins

☐ Folate

☐ Vitamin C

☐ Iron

☐ n-3 PUFAs

☐ Other: ……………………………………

https://doi.org/10.3390/xxxxx
